# Transparency by Chinese cities reduces pollution violations and improves air quality

**DOI:** 10.1073/pnas.2406761122

**Published:** 2025-04-04

**Authors:** Mengdi Liu, Mark T. Buntaine, Sarah E. Anderson, Bing Zhang

**Affiliations:** ^a^School of International Trade and Economics, University of International Business and Economics, Beijing 100029, China; ^b^School of Environmental Science & Management, University of California, Santa Barbara, CA 93106; ^c^School of International Trade and Economics, Nanjing University of Finance and Economics, Nanjing 210023, China; ^d^State Key Laboratory of Water Pollution Control and Green Resource Recycling, Nanjing University, Shanghai 200092, China

**Keywords:** transparency, air quality, environmental governance, China

## Abstract

We provide national-scale experimental evidence from China showing that transparency by local governments improves the management of air pollution. Governments that perform better have more reasons to be transparent, making the causal relationship between transparency and policy outcomes difficult to disentangle. In 2015, we randomly assigned municipal governments in China to a high-visibility, public rating of their adherence to national requirements for transparency about their regulation of pollution. By 2016, this treatment significantly boosted transparency in treated cities relative to control cities, allowing us to observe the effect of randomly increasing transparency in the years that followed. Subsequently, high-polluting firms in treated cities cut their violations by 37% compared to similar firms in control cities. Inspections by local governments increased by about 90% in treated cities relative to control cities. Ambient air pollution decreased between 8 and 10% in treated cities relative to control cities, which likely generated significant health benefits. This study provides strong evidence that governmental transparency causes improved environmental quality, at least in a setting where the public and higher governments want to hold local governments accountable.

## Significance

Governments around the world have been urged to become more transparent, because it allows the public, firms, and other governments to hold them accountable for achieving policy goals. In a national-scale experiment in China, we provide evidence that transparency by local governments increased firms’ compliance with environmental regulations and improved ambient air quality, which we calculate saved 2,008 lives per year in the treated cities and would save 24,350 lives per year across China with similar improvements to air quality. This study provides strong causal evidence about the potential for transparency to improve policy outcomes, bolstering calls for greater transparency in a variety of policy domains around the world.

Air pollution poses a grave challenge to human health across the globe, contributing significantly to mortality and morbidity. Annually, it is responsible for over 5.55 million premature deaths worldwide (1). However, as with many other areas of pressing societal concern, the crux of this issue is often not the absence of regulations. It rather stems from the failure of governments to effectively implement existing environmental regulations (2). For air pollution specifically, governments’ inability to address non-compliance has been noted for industrial emissions (3, 4), ambient air quality standards (5), crop burning (6), and vehicular emission (7).

Transparency is often promoted as a way to address failures in implementing different types of policies worldwide (8). We define transparency as the public release of information by government that is useful for evaluating the actions and performance of government. International treaties (9), international organizations (10), national governments (11), and non-governmental organizations (12) have all promoted transparency practices to improve policy outcomes.

While the ways that transparency by governments improves policy implementation likely vary by context and policy domain, the common idea is that transparency gives interested parties the information that they need to pressure governments for better outcomes, including through lawsuits (13), complaints (4), and programmatic voting (14). Transparency can activate public attention (15, 16) and facilitate the activities of nongovernmental organizations (17, 18). Transparency is also thought to facilitate political oversight between different levels of government (19) and improve policy coordination across governmental units (20). In terms of air pollution, evidence suggests that firms respond to the disclosure of pollution information and the threat of scrutiny by curtailing their emissions (21).

However, since effective governments have more incentives to adopt transparent practices, the causal relationship between transparency and policy outcomes is difficult to disentangle. Governments have more reason to be transparent when they perform well (22), when they have greater capacity (23), and when there are political demands for accountability from powerful actors (24). These factors may independently cause better policy performance, rendering any observed association between policy outcomes and transparency spurious.

We used a randomized experimental design to induce an exogenous increase in governmental transparency in China, which addresses these core challenges directly. The first stage of the experiment involved publicly rating 25 municipal governments in China on their compliance with national rules to disclose information about enterprise emissions, ambient environmental quality, inspections, and environmental impact assessments, among other topics. The ratings only pertained to whether local governments publicly disclosed the required information, not whether the information indicated good or bad environmental performance. We compiled the same ratings for 25 control cities but did not disclose them. As reported previously, the intervention significantly increased the amount of environmental information disclosed by treated cities relative to control cities (25).

Since public ratings improved transparency among a random set of local governments, we now move to the second stage of the study. Here, we examine the downstream consequences of improving transparency on the implementation of air quality standards and pollution regulations, without the usual confounding factors. While we used ratings to increase transparency, other tools or policies could achieve similar results. Our focus in this stage is the consequences of improving transparency for environmental management. Because treated cities released more information, they should be subject to more scrutiny or the threat of scrutiny than control cities, either from the public, non-governmental organizations, or the central government. If treated governments had disclosed more information but did not improve environmental management, we would conclude that transparency is unlikely to improve environmental performance. Instead, we find that the longer-term consequence of increasing governmental transparency is increased regulatory effort by local governments and improved environmental quality.

Specifically, we find that randomly increasing transparency by local governments improved ambient air quality, reduced pollution violations by industrial firms, and increased regulatory efforts. Industrial emissions (excluding power generation) accounted for 32% of PM2.5 air pollution in China in 2010, five years before the launch of our study (26). Because of their spatial distribution, these emissions were a leading cause of premature mortality (27). In treated cities that had their transparency randomly boosted, we observed a reduction in ambient air pollution by 8 to 10% relative to control cities over the next five years. This decrease in pollution levels would translate to an approximately 0.25% drop in the rate of all-cause mortality (28), saving an estimated 24,350 lives annually if it were achieved across China. High-polluting firms in treated cities had 37% fewer days with emissions violations, and governmental enforcement actions, such as inspections, increased by 90% in treated cities as compared to control cities. These findings underscore the tangible benefits of increasing transparency.

Our study is not the first to address the environmental impacts of transparency practices by local governments in China. The rating scheme that we study, the Pollution Information Transparency Index (PITI), has been studied using two types of research designs, and we build upon these results. Some studies examine how cities that have been rated by PITI differ in their pollution outcomes, green innovation, or foreign direct investment as compared to unrated cities using difference-in-difference designs (29–32). Many of these studies report positive effects of being rated by PITI on these outcomes, though the strength of these effects is disputed, since many studies do not account for concurrent administrative and policy mandates unique to local governments that were originally rated by PITI (30). Indeed, the PITI program was originally applied to cities designated by the central government as key cities for pollution control. Since the original program cities also received different targets and increased resources for addressing pollution, it is difficult to disentangle the effects of transparency from other policies without the type of research design we report here.

Other studies have examined the correlation between PITI scores and environmental outcomes in rated cities. The results are mixed with both positive (33, 34) and null (35) effects reported. These mixed results are difficult to synthesize because of different sample years and modeling choices. More fundamentally, these studies assume that governments do not select their level of transparency in light of pollution outcomes within their jurisdictions, a pattern clearly identified in previous research (22). In light of the methodological challenges and mixed results, we use an experimental research design that provides more definitive evidence with a more comprehensive set of outcomes and over a longer period of time than prior studies. Given the global promotion of transparency practices, stronger, more complete evidence is needed.

Policy failure at implementation is not exclusive to environmental problems or air pollution. Across various sectors, governments often fall short in executing policies as intended. Such lapses in government performance are well documented in health services (36), education (16), public finance (37), and infrastructure (38), among many other areas. A common diagnosis for these failures is a lack of transparency, which leaves the public and other levels of government uninformed and unable to hold governments accountable, allowing politicians and bureaucrats to shirk their responsibilities (16). Our study provides strong evidence that supports the call for greater transparency as a way to improve policy outcomes, at least when the public or other levels of government have the interest and tools to hold governments accountable for policy performance. These results have direct relevance to other countries where regulators actively encourage greater transparency as a way to ensure industry compliance with pollution rules, such as the United States (39), India (40), Canada (41), and Indonesia (42), among others.

## Research Design

### Stage 1. Rating Local Governments and Transparency.

The first stage of this research design, which we reported previously, involved a randomized treatment that encouraged city governments in China to be more transparent by releasing information about environmental quality and regulatory efforts (25). Since 2009, our non-governmental partner in this research (IPE) has used the PITI to rate local governments on their compliance with the central government’s Environmental Information Disclosure Measures (for Trial Implementation). Under these and subsequent policies, the environmental protection departments of city governments are required to publicly disclose information on environmental inspection and supervision, enterprise emissions, pollution source self-disclosure, environmental impact assessments for new projects, and the resolution of public petitions about pollution (see *SI Appendix*, section A for further details). PITI scores assess what information is publicly available on the websites of city governments, rather than environmental regulation or quality itself. At the beginning of each calendar year, PITI scores are assessed and assigned for the prior year. City governments are required to display relevant data in these categories on their websites in timely and accessible formats.^*^ However, disclosure of the required information has always been incomplete. Whether the information reflects well or poorly on the local government, it should over time increase scrutiny or the threat of scrutiny by the public, non-governmental organizations, or the central government.

Beginning in 2008, IPE scored 113 cities designated nationally as key cities for pollution control and released those scores using reports and press releases. In 2014, they added 7 more cities that had been newly designated as key cities for pollution control. Additionally, other nongovernmental organizations that partnered with IPE rated an additional 39 cities by 2014. The average PITI score (out of 100) for the cities that IPE originally rated increased from 31 in 2008 to 57 in 2019, indicating improvements but also widespread issues with compliance (43, 44). Because many cities had already been treated and could not be assigned to a control group, they were ineligible for our experimental sample.

Between 2015 and 2017, we implemented a national-scale, randomized experiment that involved bringing 50 new city governments in China into the PITI scoring process. We selected a sample of previously unrated cities most likely to improve transparency after being rated by PITI based on having low large firm dominance, high levels of budget revenue, and less dependence on central transfers (45). We did this because we were interested in studying the effects of transparency on environmental management in this second stage of the project, but this would only be possible if the first stage provided an exogenous increase in transparency. In principle, the increase in transparency we wish to study could be induced in a variety of ways, so our focus in the second stage of this study is not directly on the rating process.

**Fig. 1.**
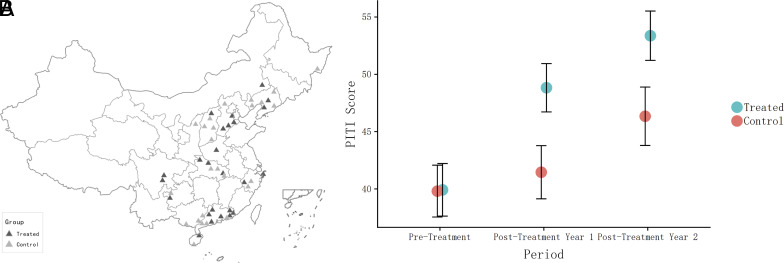
Assignment of treatment and control among experimental sample of cities and treatment effect on PITI scores. Notes: Panel (*A*) shows the distribution of 25 treatment cities and 25 control cities. Panel (*B*) shows the average aggregate scores by experimental condition in each year of the original study, with SEs derived from bootstrap sampling within experimental conditions. This figure is reproduced from ref. 25 ©2019, Midwest Political Science Association, which reported the original effects of the treatment on transparency.

The sample, shown in Panel (*A*) of Fig. 1, is mainly composed of mid-sized cities not among the most internationally well-known cities of China. The cities in our sample generally reflect the overall distribution of Gross Domestic Product per capita and population levels found in all cities in China (*SI Appendix*, Fig. S6). Additionally, our cities also have comparable levels of budget revenue and large firm dominance as all cities in China, while having somewhat lower reliance on central transfers. After selecting the sample, we formed matched-pair blocks of cities based on the pretreatment PITI scores collected for all sample cities before the start of the treatment. We randomly assigned one city in each matched pair to treatment and control.

The randomized treatment involved releasing the PITI score based on the information that local governments disclosed on their websites about enterprise emissions, inspections and supervision efforts, resolution of complaints, and environmental impact assessments, among other requirements.^†^ The scores for treated cities were released as part of IPE’s annual PITI report and scorecard, which showed how each city compares to others in meeting transparency requirements. IPE released the report and scorecards in a press release and launch event, which was covered in television and print media. We delivered the reports that contained the scores to the environmental protection bureaus at each city government in the treatment group. The control group was unaware of monitoring and never had their scores released.

As we reported previously (Fig. 1*B*), the treatment increased transparency in treated cities by approximately seven points (P<0.01) on the 0 to 100 scale in the first year and this effect persists with reinforcement into the second posttreatment year (P=0.06) (25). The PITI scores for each city before and after the experiment are depicted in *SI Appendix*, Fig. S1. Because the first stage of the research design created an exogenous increase in transparency among treated cities, we are able to proceed in this paper to the second stage of the design and follow the consequences for environmental management over several years.

### Stage 2. Transparency and Environmental Performance.

After successfully increasing transparency in the treated cities, we track the downstream effects on air quality, firm-level emissions, and government enforcement over several years. This analysis shows how randomly increasing government transparency impacts environmental performance. We estimate intent-to-treat effects, capturing the average impact of rating city governments with PITI on regulatory and environmental outcomes. Since our outcome data extend beyond the period of the transparency intervention, the main results should be seen as the longer-term effects of encouraging greater transparency. We also estimate complier average causal effects using an instrumental variables approach (*SI Appendix*, Fig. S3), which captures the short-term impacts among cities that became more transparent due to the PITI treatment.

We hypothesized that transparency would improve environmental management outcomes because it would allow the public (15), NGOs (17, 18) and national government agencies (19, 20) to seek accountability from local governments. The idea that transparency will improve governance is not a new hypothesis and it has been proposed and investigated in China and other settings (46, 47). Our study leverages increased transparency among a random set of governments on a rich set of environmental and governance outcomes realized several years later, including ambient air quality, firm-level pollution violations, and governmental enforcement activities.

The experimental design of this study helps to resolve two major challenges with empirically evaluating whether transparency improves policy implementation and environmental governance. First, it is extremely difficult to study the effects of transparency because governments that perform well have more reasons to be transparent. Cities’ efforts to be transparent may follow their efforts at reducing pollution, making correlational studies problematic for determining whether transparency improves environmental quality (33, 34, 48). Studies that use the PITI score as the independent variable and associate that score with pollution outcomes (34, 35, 49) face difficulties overcoming this core inferential challenge. A relationship between PITI scores and pollution may stem from local governments that have good environmental performance choosing to implement transparency practices. More dynamically, local governments might first improve their environmental performance, in anticipation of later improving their transparency, leading to no relationship between the variables in a particular year.

Other studies have used being rated by PITI in a difference-in-difference approach (30–32), but the original PITI rating was applied to cities that had been designated as national priorities for pollution control and therefore had been allocated different targets and resources than other cities (29). This confounds the treatment assignment and makes it difficult to assess whether being rated by PITI or being designated as a key city for pollution control by national agencies improves environmental outcomes. The bulk of studies using both research designs have found a positive relationship between transparency and environmental outcomes, though this conclusion has been challenged both substantively and methodologically (30, 35).

There are hardly ever opportunities for exogenous changes in transparency that only affect some governments under study. Our study leverages such an opportunity. Additionally, existing studies have not yet brought together an analysis of ambient air quality, firm-level pollution violations, and enforcement activities by local governments. In terms of air quality, we investigate relative changes in ambient air quality between treatment and control cities as measured by the national network of air quality monitoring stations, aggregated to the monthly level. We expect that local governments will do more to regulate all sources of pollution when they potentially face greater scrutiny due to transparency. In terms of pollution violations, we use data from firms that are part of the Continuous Emissions Monitoring System that measures hourly emissions of key pollutants at the stack level to examine whether fewer firms violate daily emissions limits in treated cities as compared to control cities. We expect that firms that regularly violate their permitted emissions standards will comply more often in treated cities. In terms of improvements to regulation, we use data on the environmental inspections conducted by local governments to compare treated to control cities. We expect that local governments will regulate high-polluting firms more stringently after those governments are prompted to adopt transparency practices (50). We also expect that firms that sell goods directly to consumers will be more likely to reduce emissions as a downstream consequence of increased transparency, because they are responding to market pressures directly (21). In each case, we use data from 2015 to 2020, which allows us to track the impact of increasing transparency on these outcomes over several years.

## Results

### Ambient Air Quality.

**Fig. 2.**
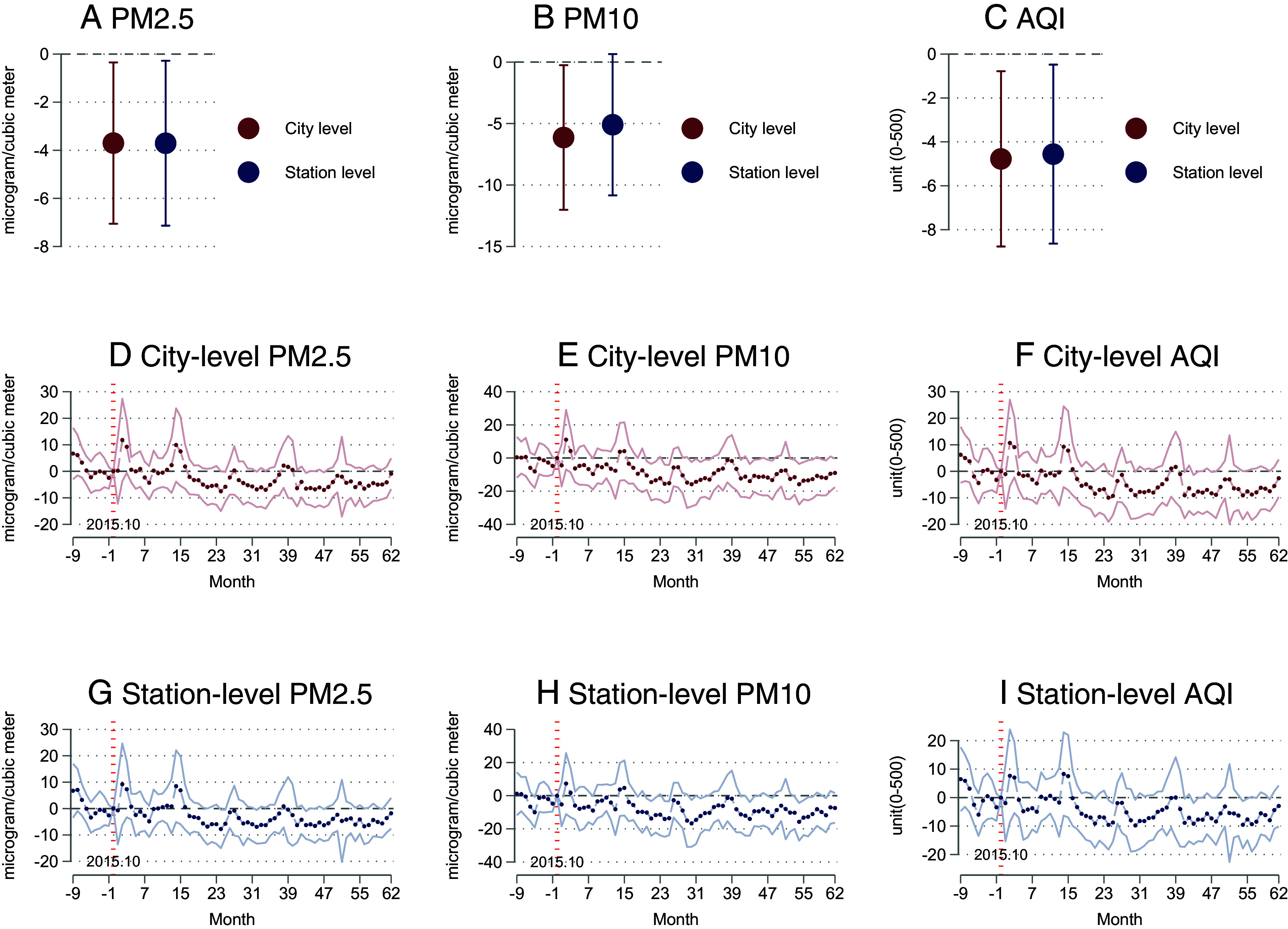
Effects of being rated by the PITI program on ambient air quality. Notes: This figure reports the impact of being rated by the PITI program on air quality from 2015 to 2020, as measured by PM2.5, PM10, and AQI. Panel (*A*) presents coefficients and 90% CIs on Treat*Post from Eq. **1** and Panels (*B* and *C*) present the dynamic DID results using the month prior to the transparency treatment as the baseline at the monitoring station and city level. Treatment began in September 2015. We use city-level data aggregated to the monthly level, control for month and city fixed effects, and cluster SEs at the city level in all specifications. Panels (*D*–*I*) show city- and station-level dynamic effects by month.

On average, being rated as part of the PITI program led to a reduction of 3.7 micrograms per cubic meter of urban PM2.5 and 6.1 micrograms per cubic meter of PM10 and a reduction of 4.8 units in the air quality index (AQI) (Fig. 2*A*–*C* and *SI Appendix*, Tables S6 and S7). The decreases account for 9.6%, 9.1%, and 7.6% of the baseline values of PM2.5, PM10, and AQI, respectively. The negligible impact observed during winter could be due to coal heating being the primary source of air pollution in the season, especially in the northern regions (51, 52). Another possibility is that during winter, all regions in China adopt more stringent air pollution control measures.^‡^ An examination of the dynamic effects indicates that it takes approximately one year after being rated by PITI for air quality to improve (Fig. 2*D*–*I*). While we show the main effects in terms of the net consequences of being rated by the PITI program, results using random assignment as an instrument for the level of transparency in a city are consistent, though more imprecise (*SI Appendix*, Fig. S3).

The city-level results on ambient air quality are robust to using satellite data to measure pollution, as indicated by PM2.5 and PM10 levels. This mitigates the concern that data from air quality monitoring stations are manipulable by the cities that are rated by the PITI program (see *SI Appendix*, Table S13 for details). These remotely sensed data are derived from a US-supported NASA satellite instrument (53, 54).

### Pollution Violations.

**Fig. 3.**
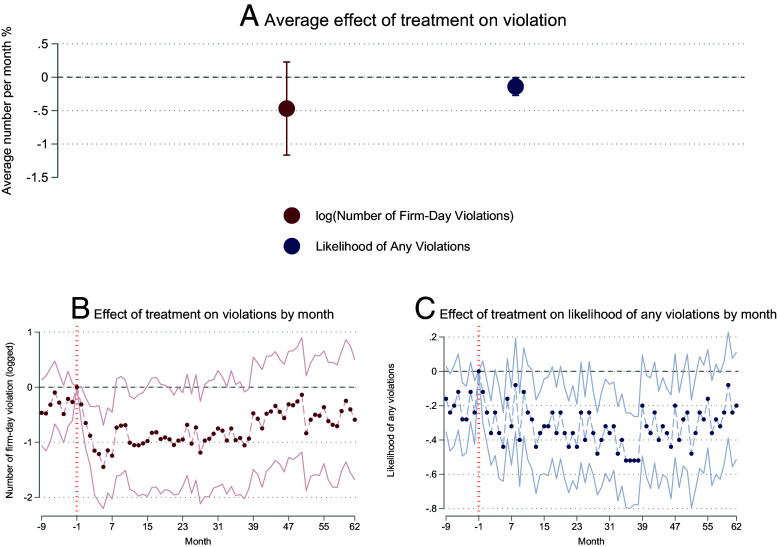
Effects of Transparency Improvement on City-level Violations. Notes: Panel (*A*) reports the DID effects of increasing transparency on city-level regulatory outcomes from 2015 to 2020, as measured by the number of violating firm-days and whether any violation occurred. Panels (*B* and *C*) show the effects of the transparency intervention broken down by month. In all regressions, we use monthly city data, control for month fixed effects and city fixed effects, and cluster SEs at the city level.

We next demonstrate that industrial firms specifically reduce pollution violations more in the treated cities. As is shown in Fig. 3*A*, being rated by the PITI program decreased the count of firm-days with violations of emissions standards by ∼37% among all firms with automatic stack-level monitoring, though this estimate is noisy. The intervention also decreases the probability that any violation by any firm occurred in a city during a given month by ∼13%. The *Bottom* two panels of Fig. 3 also demonstrate that the effects of the treatment persist for at least five years after the intervention. The results are robust to outlet-level analyses (*SI Appendix*, Table S4).

Six cities in Hubei province (three in the control group and three in the treatment group) had their PITI scores released by another non-governmental organization starting in 2019, making the intent-to-treat estimates we report a lower bound. As robustness checks, we consider the six cities in Hubei province as receiving treatment post-2019 or exclude them from our sample post-2019, which yields similar results (*SI Appendix*, Table S5). These results show that being rated by the PITI program and thereby improving transparency resulted in increased compliance with emissions standards relative to control cities.

**Fig. 4.**
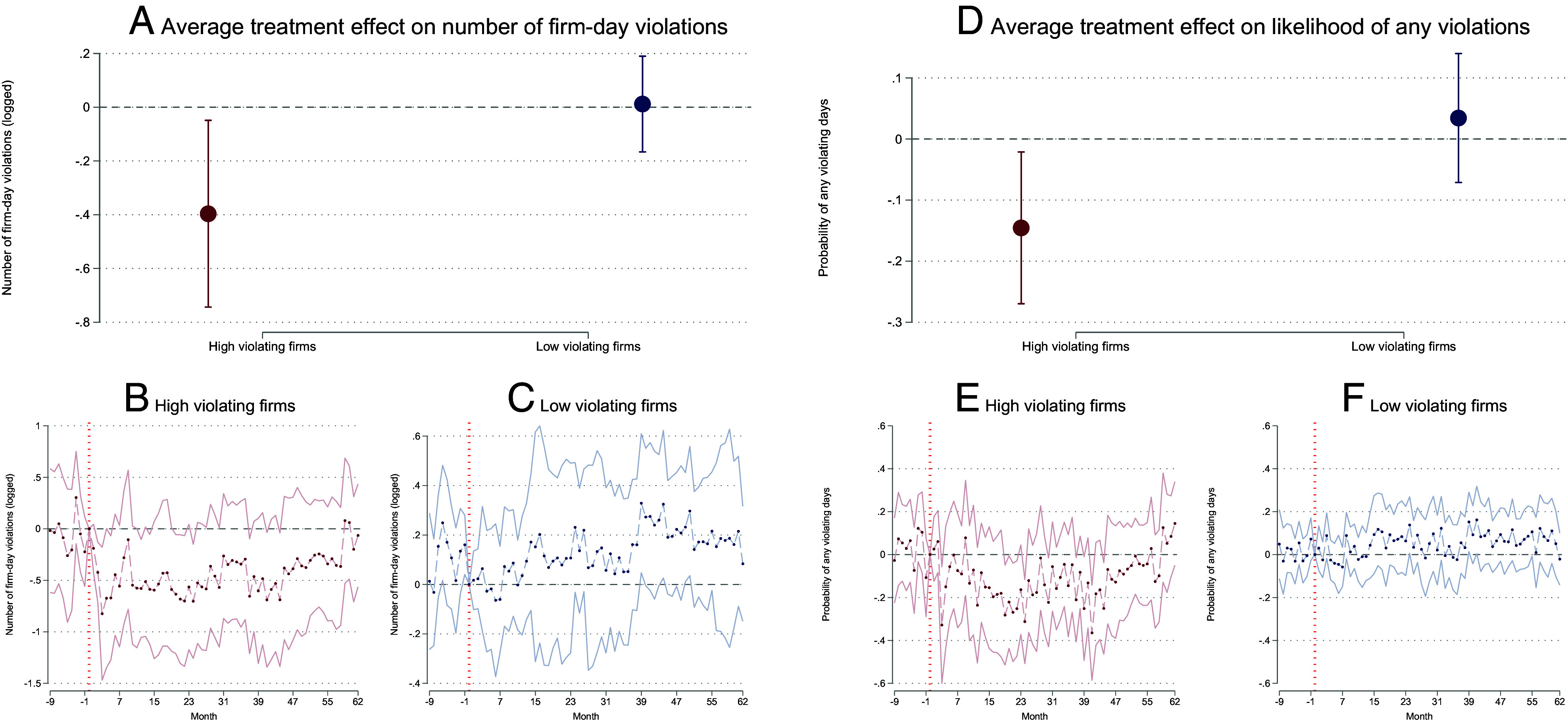
Effects of Transparency Improvement on Firm-level Violations. Notes: This figure reports the impact of being rated by the PITI program on firm-level outcomes from 2015 to 2020. Panels (*A* and *B*) report the heterogeneous DID, and panels *B*-*F* show the dynamic DID impacts on high and low polluting firms, based on benchmark violations using the top 25% as the cutoff. We use monthly city data, control for month fixed effects and firm fixed effects, and cluster SEs at the city level in all specifications.

Because they are likely subject to greater scrutiny under transparency, we expect enterprises with high baseline levels of violations to be the ones to show improvement (55). In fact, firms in the top 25% for violations in the baseline period reduced violations once transparency improved in the treated cities (Fig. 4). The treatment had little impact on the pollution of enterprises with a low rate of baseline violations. This result is robust to the choice of cutoffs (*SI Appendix*, Tables S8–S11).

In terms of the validity of these results, one concern is that polluting enterprises may manipulate automatic monitoring data in response to transparency rather than reducing emissions. We examined this possibility by comparing the frequency of abnormal readings in treated versus control firms. According to *SI Appendix*, Table S14, the transparency treatment had no effect on the probability that the Continuous Emission Monitoring System (CEMS) were operated for less than 20 h per day or the probability that CEMS recorded exceptionally low emission concentrations, defined as daily average pollutant concentrations that are less than one-tenth of the firm’s average annual concentration. These results indicate that data manipulation is unlikely to account for our primary findings.

To test whether the government strategically closes down high-polluting enterprises to show improvement, we examined the entry and exit of enterprises and found no detectable effect on firm closures, as defined by firms that stop producing hourly monitoring data (*SI Appendix*, Fig. S4). Additionally, we found that the installation of new CEMS equipment in existing firms was not related to the treatment assignment (*SI Appendix*, Fig. S5). These results indicate that the treatment effect of being rated by the PITI program results from changing production practices of firms, rather than changes in the composition of firms in the sample.

Another concern is the potential difference in the intensity of environmental policies implemented after the treatment between the treatment and control groups, which could confound our results (see *SI Appendix*, section F for further details). We addressed this by conducting balance tests on the two main environmental policies in China from 2015 to 2020. First, for the “Three-Year Action Plan to Win the Blue Sky Defense War,” which targeted reductions in PM2.5, SO_2_, NO_*x*_, and volatile organic compounds emissions through stringent controls in key regions such as Beijing-Tianjin-Hebei, the Yangtze River Delta, and the Fenwei Plain, both treated and control groups had an equal distribution of cities in key regions. Second, for the Central Environmental Protection Inspectorate initiative, which involved central government teams conducting intensive, month-long oversight visits to provincial and local governments, the distribution of cities across inspection batches showed no significant differences between the treated and control groups (*SI Appendix*, Table S16).

### Enforcement by Local Governments and Consumer Pressures As Mechanisms.

**Fig. 5.**
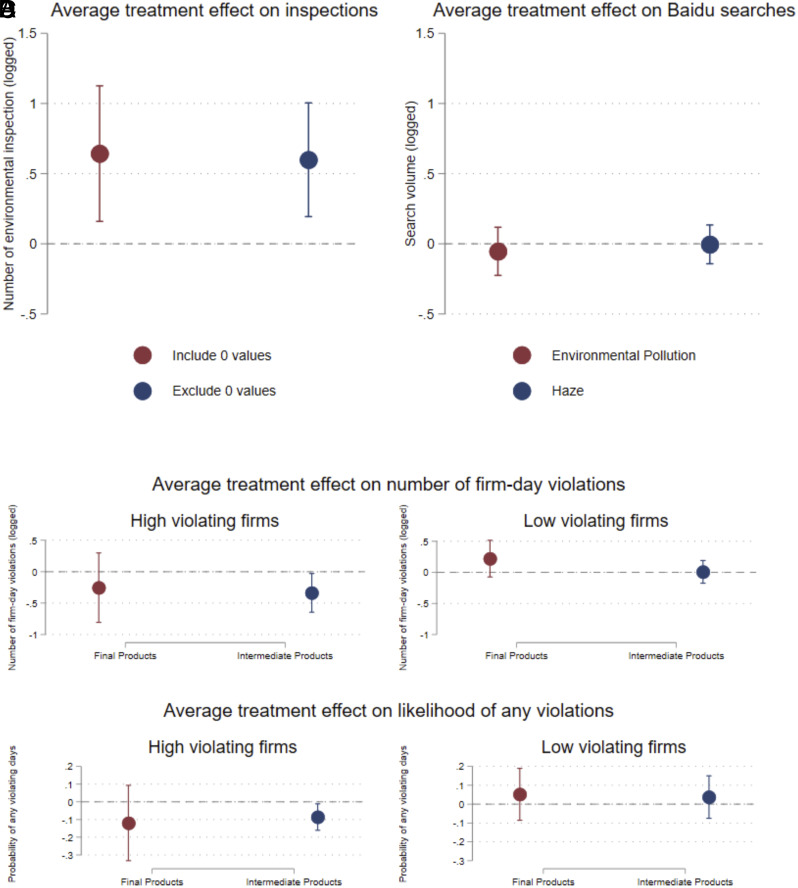
Evidence About Mechanisms for Environmental Improvements after Transparency. Notes: Panel (*A*) reports the effects of treatment on city-level environmental inspections from 2017 to 2020, as measured by the number of environmental inspections per month, for a sample that includes and excludes firms with zero inspections for the city-month. In all regressions, we use monthly city data, control for month fixed effects and block fixed effects, and cluster SEs at the city level. Panel (*B*) reports the effects of treatment on city-level Baidu searches from 2014 to 2020. For panels (*A*–*C*), we use monthly city data, control for month fixed effects and city fixed effects, and cluster SEs at the city level. Panels (*C* and *D*) report the heterogeneous DID impacts on high and low polluting firms, using firms in the top 25% of violations as the cutoff, by types of products they produce. For panels (*C* and *D*) we use firm-month data, control for month fixed effects and firm fixed effects, and cluster SEs at the city level.

Consistent with the expectation that transparency will increase regulatory stringency by city governments, Panel (*A*) of Fig. 5 and *SI Appendix*, Table S12 show that treated cities had substantially more inspections after the transparency intervention compared to control cities. Panel (*B*) of Fig. 5 shows that online searches for “environmental pollution” and “haze” on the Baidu search engine did not change, which is consistent with evidence in our prior paper that citizen and news media attention to pollution and transparency was not higher in treated cities (25). However, the lack of any detectable change in public attention must be interpreted carefully, since governments might be responding to the threat of public attention, which might not materialize when environmental quality improves. Panels (*C* and *D*) of Fig. 5 indicate that the pollution reduction did not come only from high-emitting firms that sell final goods to consumers, which should be more sensitive to direct public pressure. Taken together, these results indicate that firm reductions in emissions are more consistent with governments regulating more stringently following transparency, rather than a direct response by firms to public pressure. We are not able to measure the specific ways that firms responded to the enforcement brought about by transparency, but there is substantial evidence that firms adjust to enforcement by investing in cleaner technology, switching to cleaner fuels, or operating energy-intensive abatement equipment (56).

### Discussion and Conclusion.

Transparency has been promoted globally as a part of efforts to reduce pollution. Yet persuasive evidence that transparency improves governance and policy outcomes is relatively sparse. Transparency has been studied with observational research designs (29), but these rely on the assumption that there are no other confounding policies or factors that drive both transparency and policy outcomes. These studies show mixed results: Some find that transparency reduces pollution, possibly at the expense of neighboring cities (30, 48), while others find no effect on pollution (35). This underscores the need for experimental evidence to resolve the inferential challenges.

We create a unique, exogenous shock in transparency by city governments using a randomized controlled trial. Based on this shock, we show that improving transparency has significantly reduced pollution, likely by improving oversight of firms with a high number of violations. Assignment to treatment reduced approximately 6 emissions violations per month per city among industrial firms subject to automatic monitoring, which represents a 37% decline in violations relative to the control group. In terms of ambient air pollution measured by national sensors within city limits, the transparency treatment reduced PM2.5 by 9.6%, PM10 by 9.1%, and AQI by 7.6% relative to the control group.

Transparency that allows the public and other levels of government to gain information on firms’ emissions, ambient pollution, and what local governments are doing to address them created the conditions for improved enforcement of environmental standards in China, closing the “implementation gap” that has emerged between central policies and local implementation (57). Both firms and governments are making changes. While we do not observe the specific means by which firms are adjusting to increased transparency and regulatory effort, such as through technological innovations, fuel switching, or changes to production processes, related results suggest transparency prompts firms to innovate and patent new ways to produce goods with fewer negative environmental consequences (58). Local governments are increasing their inspections and enforcement, perhaps adjusting to the expectation of significant public attention or top–down pressure from the central government.

In terms of generalizing the results from this study to other cities in China and beyond, there are at least three relevant thought experiments. First, what would happen if we were able to work with a nongovernmental organization to rate the transparency of all cities in China using PITI, assuming that none had been rated previously? Since we argue that being rated affects environmental quality through transparency, this is mainly a question of whether the PITI rating would induce a smaller or larger transparency effect in the sample with all cities. We selected a purposeful sample of cities that had not been rated by IPE (120) or other nongovernmental organizations (39) by 2014 but that were most likely to increase transparency among the remaining cities. Specifically, we selected cities that had higher levels of budget revenue, less dependence on central transfers, and lower large-firm dominance, all of which are predicted to result in a larger effect of being rated by PITI (25, 45).

We detect no difference between the sample cities and all cities on budget revenue and large firm dominance, while the sample cities are slightly less reliant on central budget transfers than all cities (*SI Appendix*, Fig. S7). This means that our sample is likely to be slightly more responsive to the PITI rating as compared to all cities in China. However, as compared to key cities originally rated under the PITI program, which are home to about half of China’s population and 40% of industrial firms, our sample has characteristics that are likely to make it less responsive to PITI ratings (*SI Appendix*, Fig. S8). In sum, our sample estimates are likely more than what can be achieved using nongovernmental ratings in smaller, less industrialized cities, but less than what can be achieved in larger, more industrialized cities.

Second, how would environmental quality change if transparency were increased across all Chinese cities, as observed in the treated group, using ratings or other methods? To explore this, we can identify which firms in our study responded to increased transparency and determine whether these firms are more or less common in all cities across China. High-polluting firms were most likely to respond to the increase in transparency (Fig. 5*C*) and our sample of cities had more high-polluting firms than all cities in China, but fewer high-polluting firms than the larger, more industrial key cities original rating under PITI (*SI Appendix*, Fig. S6).^§^ As above, this means that the effects of transparency on pollution are likely to be lower in a population that includes many less industrialized cities and higher in a sample of more industrialized cities, especially since more industrialized cities are also under more pressure from the central government to improve environmental outcomes.^¶^

Our study largely corroborates the findings of recent observational studies that find transparency by local governments in China reduces pollution (29–31, 33, 34, 48), but its conclusions differ from earlier studies of PITI (22, 35). Recent analysis of the PITI program suggests that reductions in pollution depend on complementary career incentives for local officials to meet pollution targets (59). The effects of PITI on pollution reductions appear to have grown over time (29). Our primary contribution is to strengthen this body of evidence with a robust research design for causal inference, confirming that transparency by local governments reduces pollution in China. By experimentally manipulating governmental transparency, we rule out the possibility that transparency levels are merely a reflection of existing pollution-control efforts. Additionally, we address confounding issues from prior studies, as PITI was initially implemented in cities that also received special targets and resources for pollution control. Our study uses a different sample than prior studies, though this should have attenuated pollution reductions, since our sample of cities has less heavy industry than key cities that were the focus of prior studies. Overall, our study adds strong, causal evidence to this growing literature, bolstering confidence in recent findings, particularly since our sample is less likely to respond as compared to the cities originally rated by PITI, which have been the focus of prior studies.

Finally, could similar increases in transparency improve outcomes in other settings or policy areas? In the case of pollution in China, both the public (4) and the central government (60) have strong interests in improving environmental quality. Transparency is valuable when it provides information that enables interested parties to hold governments accountable, through mechanisms such as complaints, lawsuits, voting, or oversight. The ability of interested parties to use disclosed information to seek accountability in these ways will vary by context, so a uniform outcome of transparency is unlikely. We hypothesize that transparency will have the strongest effect in contexts with high public and governmental interest, such as environmental quality. In these situations, both the public and officials are motivated to act on disclosed information. Fundamentally, this means that information must be released in a way that enables better evaluation of government performance. Additionally, it must be provided in a setting where the public, advocacy groups, or other officials have the interest and ability to hold governments accountable for performance. The link between transparency and policy outcomes is likely broken if either the information released is not useful for evaluation or interested groups are not available or cannot seek accountability in these ways. Reviews of transparency in other domains have identified similar links between transparency and policy outcomes (61). Regulators of industrial pollution have embraced a strategy of transparency across many other countries, such as the United States (39), India (40), Canada (41), and Indonesia (42), suggesting a broad belief that these supportive conditions are common.

Air pollution poses significant threats to human health, especially in rapidly expanding economies like China. In China, air pollution caused 1.24 million deaths in 2017, including 851,660 deaths from ambient PM2.5 pollution (62). Several studies have investigated the effects of various Chinese environmental regulations on PM2.5 reduction and health benefits (63, 64). Multiplying the amount of pollution reduced as a consequence of the transparency intervention in this experiment with estimates of the likely health consequences of reducing air pollution (28) yields approximately a 0.25% reduction in all-cause daily mortality, which equals 2,008 avoided deaths in the 25 treated cities each year. If similar improvements to air quality were achieved across China, there would be 24,350 deaths avoided each year (*SI Appendix*, section H).

## Materials and Methods

### Data and Outcomes.

We measure the primary environmental outcome using city-level ambient air quality data from 2015 to 2020. We specifically use pollution concentrations of PM2.5 and PM10, as well as an aggregate (AQI; see *SI Appendix*, section B for more descriptions) as measured and released in real-time by 1,563 monitoring stations across the country. On average, each city in our sample has four monitoring stations and we use average values of all monitoring stations within each sample city. Panels (*A* and *B*) of *SI Appendix*, Fig. S2 show that the average annual pollution concentration of both the treatment group and the control group has dropped over time, but the decline in the cities assigned to treatment has been more pronounced.

We measure firm-level pollution emissions using data from China’s CEMS. In 2014, the central government of China created the National Specially Monitored Firms pilot program, mandating that major industrial polluters responsible for 65% of total emissions install automated monitoring equipment that measures and uploads their emission data from each outlet in real time to the central environmental protection department and makes it public on municipal and provincial websites. Firms are ordered by their total pollution and included in the scheme if they are in the group that makes up 65% of total pollution. On average, 33 major industrial polluters are monitored for SO_2_, NO_*x*_, and dust (total suspended particulates) in each city in our sample. The data contain information regarding pollutant concentration and emission standards at outlet (typically smokestack, boiler, or machine) and firm levels, which we use to identify pollution violations among key firms in each city of our sample.

We aggregate the underlying hourly or daily data from these cities into monthly averages of the total number of firm-days with violations of emissions standards, the total number of outlet-days with violations of emissions standards for each city, and the daily average concentrations of PM2.5, PM10, and AQI. Further details on measurement are available in *SI Appendix*, section B. Panels (*C* and *D*) of *SI Appendix*, Fig. S2 illustrate that the number of cities with violations and the violation rates per city declined over time in both groups. However, the treatment group experienced a larger decrease.

We measure enforcement by local governments by extracting information from the Bureau of Ecological and Environmental Enforcement’s regulatory enforcement platform for each inspection conducted by local environmental protection departments. The Enforcement Information Platform provides reliable real-time inspection information starting from 2017. This includes details such as the name and location of the inspected firm, the start and finish times of each inspection, the type of enforcement undertaken, and a transcript summarizing the decisions made during enforcement actions. We aggregate the detailed inspection data into monthly totals for each city.

We categorize firms into those producing intermediate products and those producing final products based on the industry and business scope in the business registration data, which is from China’s State Administration for Market Regulation (4).^#^ Specifically, firms producing final products include those whose business scope contains keywords such as “beverages,” “food,” “catering,” “clothing,” “alcohol,” and “dairy products.”

### Analysis Methods.

For the main analysis, we employ the difference-in-differences (DID) method to estimate the impact of the randomized transparency intervention on pollution violations and ambient air quality between 2015 and 2020. Specifically, we estimate:

[1] $$Y_{\mathrm{it}}=\beta Treat_{i}\times Post_{t}+\theta_{t}+\alpha_{i}+\epsilon_{\mathrm{it}},$$

where $Y_{\mathrm{it}}$ is the environmental or regulatory outcome in city i in month t, $Treat_{i}$ represents whether the city is in the treatment group; $Post_{t}$ represents whether the month is after the first release of PITI score as part of the treatment after September 2015. $\theta_{t}$ and $\alpha_{i}$ are month and city fixed effect respectively. We cluster SEs at the city level. For many specifications, we replace $Post_{t}$ with indicator variables for the number of months since the start of the experiment to estimate dynamic effects.

To determine whether improvement in environmental performance because of governmental transparency is driven by firms with a history of frequent violations, we construct the following model using firm-level data:

[2] $$\begin{array}{cc} Y_{\mathrm{jt}} & =\beta_{1}High_{j}\times Treat_{j}\times Post_{t}\\ & \;+\beta_{2}Treat_{j}\times Post_{t}\\ & \;+\beta_{3}High_{j}\times Post_{t}\\ & \;+\theta_{t}+\alpha_{j}+\epsilon_{\mathrm{jt}}, \end{array}$$

where $Y_{\mathrm{jt}}$ is environmental performance of firm j in month t, measured by the number of times in a month that the firm’s pollution exceeds their emission standard; $Treat_{j}$ represents whether the firm is located in a treatment city; $Post_{t}$ represent whether the month is after the first release of PITI score as part of the treatment after September 2015; $High_{j}$ represents whether the firm has a high level of benchmark violations. $\theta_{t}$ are time fixed effects and $\alpha_{j}$ are firm fixed effects. We cluster the SEs at the city level. We define high violation firms as those in the top 25%. As a robustness check, we also define high and low violating firms based on alternative cutoffs (top 10% and above mean of benchmark violating firms).

## Supplementary Material

Appendix 01 (PDF)

## Data Availability

Data and code needed to reproduce analyses have been deposited in Open Science Framework Foundation (65). Previously published data were used for this work (66).

## Footnotes

^*^In the Chinese context, city governments are prefecture-level administrations that govern cities and their surrounding areas. They are typically subordinate to provincial governments, except for four very large cities that have provincial status (e.g., Beijing, Shanghai).

^†^Though random assignment ensures that the two groups of cities are similar in expectation, tests displayed in *SI Appendix*, Table S15 on their characteristics confirms balance.

^‡^The Chinese Ministry of Ecology and Environment has implemented specific policies to strengthen industrial pollution control during the winter months, particularly in response to the air quality degradation caused by coal heating in Northern regions. A notable policy is the “Staggered Production Policy,” part of the broader “Air Pollution Prevention and Control Action Plan” initiated in 2013. This policy targets the Beijing-Tianjin-Hebei area and surrounding regions to reduce emissions from industrial activities during the winter season by mandating reduced production, suspension, or limitations in heavy pollution industries such as steel, petrochemicals, and cement.

^§^The number of high-polluting firms is significantly higher in the 113 key cities. Specifically, in 2014, there were 102 firms with SO2 emissions over 50 kg, 64 firms with emissions over 100 kg, and 43 firms with emissions over 200 kg in these key cities. In contrast, the corresponding numbers for all cities were 71, 45, and 29, and for the sample cities were 79, 48, and 30.

^¶^These projections rest on a number of assumptions, such as transparency having a constant effect on pollution regardless of the baseline level of transparency.

^#^The data on firms under CEMS from State Administration for Market Regulation were purchased from TianYanCha, which is a comprehensive database that compiles various primary sources, including the National Enterprise Credit Information Publicity System, maintained by China’s State Administration for Market Regulation. The original data can be purchased from their website (www.tianyancha.com).
