# Supplementary material for: Transparency by Chinese cities reduces pollution violations and improves air quality
Source: Proc Natl Acad Sci U S A. 2025 Apr 4;122(14):e2406761122. doi: 10.1073/pnas.2406761122 (PMC12002021; doi:10.1073/pnas.2406761122)
Supplement: Supplementary file 1 — Appendix 01 (PDF) [file pnas.2406761122.sapp.pdf]

Supporting Information for

Transparency by Chinese Cities Reduces Pollution Violations and Improves Air Quality

Mengdi LIU, Mark T. BUNTAINE, Sarah E. ANDERSON, and Bing ZHANG

This PDF file includes:

Supporting text
Figs. S1 to S8
Tables S1 to S16
SI References

Supporting Information Text

A. China's Environmental Information Disclosure

Table S1. Main policies affecting transparency in China

Time	Policy	Main contents
2002	Cleaner Production Promotion Law of the People's Republic of China (2012 Amendment)	The relevant departments of the State Council and the people's governments of provinces, autonomous regions, and municipalities directly under the Central Government shall organize and support the establishment of information systems and technical consulting service systems for cleaner production to provide the general public with methods and technologies for cleaner production as well as information and services regarding the demand and supply of recyclable waste and cleaner production policies.
2002	Law of the People's Republic of China on Environmental Impact Assessment (2018 Amendment)	The state shall strengthen the construction of the basic databases for the appraisal of environmental impacts and the system of indicators for appraisal, encourage and support the scientific research of the methods and technical specifications for appraising environmental impacts, and build a system for sharing the information about environmental impacts so as to make the environmental impact appraisals more scientific. The drafting organ shall take the opinions of the relevant entities, experts and the general public about the draft report of environmental impacts into careful consideration, and shall attach a remark whether the opinions are adopted or refused to the report of environmental impacts to be submitted for examination and approval.
2007	Regulation of the People's Republic of China on the Disclosure of Government Information [Revised]	The people's governments at or above the county level and their departments shall determine the specific government information to be voluntarily disclosed and lay stress on the disclosure of the following government information: ...Situation on the supervision and inspection of environmental protection, public health, safe production, food and drugs and product quality.
2008	Measures for the Disclosure of Environmental Information (for Trial Implementation) [Expired]	Environmental protection departments shall, within their scope of responsibilities and authorization, disclose on their own the following initiative government environmental information to the public: laws, regulations, rules, standards and other regulatory documents with respect to environmental protection; environmental protection plans; environmental quality; environmental statistics and environmental survey information; emergency plan, solid waste for large- and medium-sized cities; environmental impact assessment; pollutant discharge fees; petition letters and complaints; environmental administrative penalties; a list of the names of enterprises with serious pollution; inspection and approval results of environmental protection projects.
2012	Ambient Air Quality Standards	The policy sets PM2.5 concentration limits for both the 24-hour average and the annual mean value. The environmental protection departments are responsible for publishing the real-time hourly concentration values, daily average concentration values, AQI index and the representative area of the monitoring point for six monitoring indicators including SO2, NO2, PM10, PM2.5, O3 and CO.
2013	Measures for the Self-Monitoring and Information Disclosure by the Enterprises subject to Intensive Monitoring and Control of the State (for Trial Implementation)	The contents of self-monitoring by enterprises shall include: water pollutant discharge monitoring; air pollutant discharge monitoring; monitoring of environmental noise within the boundary of an industrial enterprise; surrounding environment quality monitoring as required by environmental impact assessment reports (tables) and the approval documents thereof.
2013	Measures for the Pollution Sources Supervisory Monitoring and Information Disclosure by the Enterprises subject to Intensive Monitoring and Control of the State (for Trial Implementation)	Environmental protection departments at all levels are responsible for disclosing to the public the supervisory monitoring information on pollution sources of key state-controlled enterprises completed at the corresponding level and at the lower level. The public information mainly includes: (1) Supervisory monitoring results of pollution sources, including: name of pollution source, location, name of monitoring site, monitoring date, name of monitoring indicators, concentration of monitoring indicators, emission standard limits, and evaluation conclusions based on monitoring indicators; (2) The reasons for not carrying out supervisory monitoring of pollution sources; (3) Annual reports on supervisory monitoring of key state-controlled enterprises.
2013	Air Pollution Prevention and Control Action Plan (APPCAP)	Prefecture-level and above cities must promptly release air quality monitoring information through major local media outlets. Environmental protection departments at all levels and enterprises must proactively disclose environmental information such as environmental impact assessments of new projects, pollutant emissions from enterprises, and the operation status of pollution control facilities, allowing public supervision. For construction projects involving public interests, public opinion should be fully considered. A mandatory disclosure system for environmental information in heavily polluting industries shall be established.
2013	Guidelines for the Disclosure of Government Information for Environmental Impact Assessment of Construction Projects (for Trial Implementation)	The environmental protection department shall disclose the following information when performing the examination and approval of environmental impact assessment documents: relevant laws, regulations, rules and management procedures for environmental impact assessment; examination and approval of environmental impact assessment of construction projects; environmental protection acceptance of completion of construction projects; qualification management of environmental impact assessment of construction projects information.
2013	Enterprise Environmental Credit Evaluation Measures (Trial)	The environmental protection department conducts a credit evaluation on the environmental behavior of the enterprise according to the information on the environmental behavior of the enterprise and in accordance with the prescribed indicators, methods and procedures, determines the credit rating, and discloses it to the public for public supervision.

2015	The Revised Environmental Protection Law of the People's Republic of China	China's legislature amended the Environmental Protection Law to include "Information Disclosure and Public Participation," making it one of only six chapters of the law. The national environmental protection department is responsible for publishing the national environmental quality, monitoring information on key pollution sources and other major environmental information. Environmental protection departments at or above the county level and other departments responsible for environmental protection supervision and management shall disclose information on environmental quality, environmental monitoring, environmental emergencies, and environmental administrative licensing, administrative penalties, and collection and use of pollutant discharge fees in accordance with the law. Key pollutant discharging units shall disclose to the public the names of their main pollutants, discharge methods, discharge concentration and total amount, conditions of excessive discharge, as well as the construction and operation of pollution prevention and control facilities. The department responsible for examining and approving the environmental impact assessment documents of the construction project shall, after receiving the environmental impact report of the construction project, disclose the full text of the report. Citizens, legal persons and other organizations have the right to report to the environmental protection department or any other department responsible for environmental protection supervision and management if they discover that any unit or individual is polluting the environment and destroying the ecology.
2015	Measures for the Disclosure of Environmental Information by Enterprises and Public Institutions [Expired]	Key pollutant discharge units shall disclose the following information: basic information, including unit name, organization code, legal representative, production address, contact information, and the main content, products and scale of production, operation and management services; pollutant discharge information, including the names of major pollutants and characteristic pollutants, discharge methods, number and distribution of discharge outlets, discharge concentration and total amount, situation of exceeding the standard, as well as the pollutant discharge standards implemented, and the approved total discharge amount; the construction and operation of pollution prevention and control facilities; environmental impact assessment of construction projects and other environmental protection administrative licenses; contingency plans for environmental emergencies.
2019	Measures for the Implementation of Government Information Disclosure of the Ministry of Ecology and Environment	Government information disclosure shall take the form of active disclosure and disclosure upon application. The policy stipulates the procedures and channels for the disclosure of environmental information, including the government website of the Ministry of Ecology and Environment, the website of the National Nuclear Safety Administration; the Bulletin of the Ministry of Ecology and Environment, China Environment News; press conferences; Ministry of government website client; radio, television, newspapers and other news media; information bulletin boards, electronic information screens, data review rooms, administrative examination and approval halls and other facilities and places; other forms that are convenient for the public to obtain information.
2022	Measures for the Administration of Environmental Information Disclosure by Enterprises	The companies affected by the Measures mainly involve those with "a high environmental impact and receive a high level of public attention". The yearly report must contain the following information: basic company information; environmental management; production, management, and discharge of pollutants; carbon emissions; ecological and environmental emergency response mechanisms; environmental violations; any other ad hoc environmental information legally required to be disclosed from the current year; any other environmental information stipulated by laws and regulations. Companies that are required to undergo mandatory clean production audits must also disclose the following information: the reason for having to undergo mandatory clean production audits; the implementation, evaluation, and inspection results of mandatory clean production audits. Both publicly listed companies and companies that issue bonds that have raised funds through the issuance of stocks, bonds, depositary receipts, medium-term notes, short-term bonds, ultra short-term bonds, asset securitization, and bank loans must disclose additional information, including: the annual financing format amount and investment targets; information on climate change and ecological and environmental protection of the projects invested by the financing.

B. AQI Description

According to the Ambient Air Quality Standards (GB3095-2012) and Technical Regulations for Ambient Air Quality Index (AQI) (Trial; HJ 633-2012) issued by the former Ministry of Environmental Protection of China, AQI includes SO_2, NO_2, O_3, PM2.5, PM10, and CO.

First, the Individual Air Quality Index (IAQI) for each pollutant is calculated using the following equation and concentration limits table.

$$IAQI_i = \frac{IAQI_{Hi} - IAQI_{Li}}{BP_{Hi} - BP_{Li}}(C_i - BP_{Li}) + IAQI_{Li}$$

where $IAQI$ is the individual air quality index of pollutant i. C_i is the pollution concentration of pollutant i. BP_{Hi} is the value in the table that is closest to C_i and larger than C_i and BP_{Li} is the value in the table that is closest to C_i and smaller than C_i. $IAQI_{Hi}$ and $IAQI_{Li}$ are the AQIs corresponding to BP_{Hi} and BP_{Li} in the table.

Table S2. Calculation of Air Quality Index

IAQI	24-hour average SO2	1-hour average SO2	24-hour average NO2	1-hour average NO2	24-hour average PM10	24-hour average CO	1-hour average CO	1-hour average O3	8-hour average O3	24-hour average PM2.5
0	0	0	0	0	0	0	0	0	0	0
50	50	150	40	100	50	2	5	160	100	35
100	150	500	80	200	150	4	10	200	160	75
150	475	650	180	700	250	14	35	300	215	115
200	800	800	280	1200	350	24	60	400	265	150
300	1600	-	565	2340	420	36	90	800	800	250
400	2100	-	750	3090	500	48	120	1000	-	350
500	2620	-	940	3840	600	60	150	1200	-	500

Notes: The units of SO_2, NO_2, PM10, O_3, and PM2.5 concentrations are $\mu g/m^3$ and the unit of CO concentration is mg/m^3.

Second, the AQI is determined by the highest IAQI, where $AQI = max\{IAQI_i\}$. The maximum IAQI pollutant is referred to as primary pollution.

C. PITI Details

The following section contains a verbatim copy of the description of the rating data used as part of the treatment. We reported our procedures previously and copy them here for the convenience of readers (1).

The Pollution Information Transparency Index (PITI) has been published since 2008 and the last report prior to our study in 2014 covered 120 core cities, with another 28 cities rated by local NGOs. The cities that were included in PITI prior to our study were purposefully chosen because they were cities designated for environmental protection in the 11th and 12th national Five Year Plans, cities designated for tourism, provincial capitals, and large cities of national importance, or evaluated by other local NGOs. Because PITI had already rated these cities, they were not eligible to be part of our experimental sample.

PITI scores cities on their information disclosure related to pollutants and pollution sources, with a total possible score of 100 points. PITI provides both aggregate and component ratings of the transparency practices of cities. Each city is evaluated in four main categories: Environmental Supervision Information (50 points), Responsiveness (15 points), Enterprise Emission Data (20 points), and Environmental Impact Assessment Information (15 points). The relative importance of each category is reflected in the points allocated. To ensure quality, when PITI was first launched, IPE solicited the opinions of a panel of experts in environmental protection, law, statistics, and other fields, whose feedback was incorporated into the evaluation methodology. In addition, IPE carried out a sensitivity analysis to assess how the rankings would be affected by different component weightings and scoring criteria. This sensitivity analysis showed a low level of sensitivity; the adjustment of weightings did not significantly affect the overall order of the ranking (IPE 2008).

All the components of PITI score are set according to China's current environmental laws and regulations, including the Regulations of the People's Republic of China on Open Government Information and the Ministry of Environmental Protection Measures on Open Environmental Information (Trial), Measures on Self-Monitoring and Information Disclosure of Key State-Monitored Enterprises (Trial), and Measures for Key State-Monitored Enterprise Supervisory Monitoring and Information Disclosure (Trial). IPE sets criteria based on the laws and regulations and quantifies the compliance of local governments with legal requirements. Since most cities have adopted limited transparency practices and no cities in existing PITI releases approach the perfect transparency score, we are not concerned with ceiling effects.

We collected official data related to transparency by municipal governments in the area of pollution sources from January 1st 2014 to December 31st 2014 (pre-treatment) and then from January 1st 2015 to December 31st 2015 (Year 1 post-treatment) and January 1st 2016 to December 31st 2016 (Year 2 post-treatment), mainly from online data sources, including Environmental Protection Bureau websites, municipal government websites, and new media platforms. IPE publishes a detailed methodology of the scoring process (IPE 2016), which enabled us to replicate the IPE scoring for the cities in the experimental sample.

Supervision Records.

Records of Enterprise Violations (23 pts): This category rates the disclosure of the municipal Environmental Protection Bureau's (EPB) monitoring of pollution sources and the publication of these monitoring results, particularly the disclosure of data regarding excessive emissions from polluters. Other types of records of facility violations, including administrative penalties, reports on enforcement actions, supervisory notices urging violators to come into compliance within a given time frame, are also included.

The 23 pts can be divided into 4 parts: systematicness (7 pts), timeliness (4 pts), completeness (8 pts), and user-friendliness (4 pts). The level of systematicness is measured by the ratio of the number of violation records disclosed by the local EPBs (b) to the amounts of violation records that are supposed to be disclosed (a), which is calculated based on average ratio of pollution emission and violations in the 20 largest cities (e.g., 7 pts if b/a is larger or equal to 1; 6.3 pts if b/a is larger or equal to 8/9 and smaller than 1; etc.). The level of timeliness is determined by how often the violation information was published (e.g., 4 pts if daily; 3.2 pts if monthly; etc.). The level of completeness depends on how much critical information is available (e.g., +1.6 pts if there is the place and the time of the violation; +1.6 pts if there are specific regulations or laws that the firm violated and specific emission standards; +1.6 pts if information about the concentration of contaminants is released). The level of user-friendliness is determined by whether the violation information is convenient for the public to access (e.g., +0.8 pts if there is a website search engine; +0.8 pts if there is a page or column specifically for violation information).

Principal Laws or Regulations: Measures on Open Environmental Information (Trial); Measures on Self-Monitoring and Information Disclosure of Key State Monitored Enterprises (Trial), and Measures for Key State-monitored Enterprise Supervisory Monitoring and Information Disclosure (Trial); Notification Concerning the Reinforcement of Pollution Source Environmental Supervisory Information Disclosure.

Enterprise Environmental Behavior (5 pts): This category rates whether the municipal government disclosed gradings for enterprises (such as the color-coded rating: very good "green", good "blue", warning "yellow", bad "red") based on pollution control, environmental compliance, and violations.

The 5 pts can be divided into 4 parts: systematicness (2 pts), timeliness (1 pts), completeness (1 pts), and user-friendliness (1 pts). The level of systematicness is measured by the ratio of the number of bad firms (coded as red) disclosed (b) to the number of bad firms that are supposed to be disclosed (a), which is calculated based on average ratio of pollution emission and firm gradings in the 20 largest cities (e.g., 2 pts if b/a is larger or equal to 1; 1.6 pts if b/a is larger of equal to 8/9 and smaller than 1). The level of timeliness is according to the time difference between the publication and creation of the firm ratings (e.g., 1 pts if the document is published within one month after the document is formed; 0.8 pts if the time difference is longer than one month and within three months). The completeness is measured by how much information about the color rating is

published (e.g., +0.4 pts if there is firm name and color; +0.2 pts if the definition of the color is consistent with the laws and regulations). The level of user-friendliness is determined by whether the color rating information is provided in a format that is convenient for the public (e.g., +0.2 pts if there is a website search engine; +0.6 pts if there is a column or page specifically for this information).

Principal Laws or Regulations: Opinion on Accelerating the Implementation of the Enterprise Environmental Performance Assessment System; Enterprise Environmental Credit Evaluation Measures (Trial); Notification Concerning the Reinforcement of Pollution Source Environmental Supervisory Information Disclosure.

Discharge Fee Data (2 pts): This category rates the disclosure of discharge fees levied against polluters, including the basis for such fees, standards and procedures for levying fees, fees owed compared to actual fees gathered, and any waivers or discounts granted to facilities.

The 2 pts are divided into 4 parts: systematicness (0.5 pts), timeliness (0.5 pts), completeness (0.5 pts), and user-friendliness (0.5 pts). The level of systematicness is determined by how many months are covered during the evaluation period (e.g., 0.5 pts if 12 months; 0.4 pts if 9-11 months). The level timeliness is according to the time difference between creation and publication of the information (e.g., 0.5 pts if the document is published within 20 days after the document is formed; 0.4 pts if the time difference is longer than 20 days and within three months). The level of completeness depends on how much critical information is available (e.g., +0.1 pts if there is firm name; +0.1 pts if there is emission concentration). The level of user-friendliness is determined by whether the discharge fee information is provided convenient for the public (e.g. +0.1 pts if there is a website search engine; +0.3 pts if there is a column or page specifically for this information).

Principal Laws or Regulations: Measures on Open Environmental Information (Trial); Notification Concerning the Reinforcement of Pollution Source Environmental Supervisory Information Disclosure.

Automatic Monitoring of Pollution Sources (20 pts): This category rates the disclosure of the total volume of effluent emissions into air and water, pollution concentrations, applicable emission limit, as well as the status of compliance through provincial-level EPB self-monitoring platforms.

The 20 pts are divided into 4 parts: systematicness (5 pts), timeliness (5 pts), completeness (5 pts), and user-friendliness (5 pts). The level of systematicness is measured by the ratio of the total volume of monitoring data disclosed on the self-monitoring platforms in December (b) to the amounts of monitoring data that are supposed to be disclosed in December (a), which is calculated based on the Measures on Self-Monitoring and Information Disclosure of Key State Monitored Enterprises (Trial), and Measures for Key State-monitored Enterprise Supervisory Monitoring and Information Disclosure (Trial) (e.g., 5 pts if b/a is larger or equal to 0.8; 4 pts if b/a is larger or equal to 0.6 and smaller than 0.8, etc.). The level of timeliness is based on the frequency of the publication of monitoring data (e.g., 5 pts if within 2 hours; 4 pts if longer than 2 hours and within 4 hours, etc.). The completeness is a measure of how much critical information is disclosed on the self-monitoring platforms (e.g., +1 pts if there is monitoring time and monitoring point; +1 pts if there is concentration information for major pollutants, including COD, SO2; etc.). The level of user-friendliness is determined by whether the monitoring information is provided is provided in a format that is convenient for the public (e.g., +1 pts if there is a provincial level platform; +1 pts if there is a map to show the locations of firms; etc.).

Principal Laws or Regulations: Measures on Self-Monitoring and Information Disclosure of Key State Monitored Enterprises (Trial), and Measures for Key State-monitored Enterprise Supervisory Monitoring and Information Disclosure (Trial); Notification Concerning the Reinforcement of Pollution Source Environmental Supervisory Information Disclosure.

Responsiveness.

Verified Petitions and Complaints (7 pts): This category rates the disclosure of information on the handling of environmental petitions and complaints received by EPBs and their resolution, including the subject of the petitions and complaints, the object of the complaint (the enterprise), whether or not the case has been accepted by the EPB, the status of the investigation, and the disclosure of any resolution.

The 7 pts are divided into 4 parts: systematicness (3 pts), timeliness (1 pts), completeness (2 pts), and user-friendliness (1 pts). The level of systematicness is determined by how many months are covered (e.g., 3 pts if 12 months; 2.4 pts if 9-11 months). The level timeliness measures the time difference between the creation and publication of the relevant document (e.g., 1 pts if the document is published within 20 days after the document is created; 0.8 pts if the time difference is longer than 20 days and within three months; etc.). The completeness is a measure of how much critical information the local EPBs publish (e.g. +0.4 if the complaint letter is published; +0.4 if the status of petitions or complaints are disclosed). The level of user-friendliness is determined by whether the information on the handling of environmental petitions and complaints is provided in a format that is convenient for the public (e.g., +0.2 pts if there is a website search engine; +0.6 pts if there is a column or page specifically for this information).

Principal Laws or Regulations: Notification Concerning the Reinforcement of Pollution Source Environmental Supervisory Information Disclosure; Measures on Open Environmental Information (Trial).

Response to Public Information Requests (8 pts): This category rates response to public information requests and whether the local environmental protection bureau has established a standard and comprehensive system for responding to public information requests, including disclosure of information regarding request procedures, provision of accurate contact information, the establishment of special offices or personnel for handling public information requests, standard and timely response to requests, and efforts to improve public convenience in making information requests.

The 8 pts are divided into 4 parts: systematicness (2 pts), timeliness (1 pts), completeness (4 pts), and user-friendliness (1 pts). The level of systematicness is mainly determined by whether the local EPBs have set up a well-developed response system and give a complete reply (e.g., 2 pts if all the two requests are replied; 1.2 pts if only parts of the requests are replied). The timeliness is based on how long the local EPBs provide requested information (e.g., 1 pts is within 15 days; 0.8 pts if 16 days; etc.). The completeness is a measure of how much critical information the local EPBs provide in their responses (e.g., 4 pts if all the information are provided in detail; 3.2 pts if all the information receives a general reply in the form of a simple list; etc.). The level of user-friendliness is determined by whether it is easy for the public to make a request (e.g., +0.2 pts if there is an online platform; +0.2 pts if the telephone number is provided; etc.).

Principal Laws or Regulations: Measures on Open Environmental Information (Trial).

Enterprise Emission Data.

Key Enterprise Emission Data (16 pts): This category rates the disclosure of key enterprises' annual report and the quality of discharge information in the report, including annual pollutant emission, hazardous waste disposal, among other areas.

The 16 pts are divided into 4 parts: systematicness (4 pts), timeliness (2 pts), completeness (6 pts), and user-friendliness (4 pts). The level of systematicness is determined by how many firms publish their annual report (e.g., 4 pts if more than 80%; 3.2 pts if more than 40% and less than 80%; etc.). The level of timeliness is based on when the annual reports are published (e.g., 2 pts if early than January 31; 1.6 pts if postponed for one week; etc.). The completeness is a measure of how much critical information is in the annual reports (e.g., +1.2 pts if there is water or air emissions data; +1.2 pts if there is hazardous waste information; etc.). The level of user-friendliness is determined by whether it is easy for the public to find the annual reports (e.g., +1.6 if local EPBs construct a platform; +1.6 if the local EPBs share the information with media; etc.).

Principal Laws or Regulations: Measures on Self-Monitoring and Information Disclosure of Key State Monitored Enterprises (Trial), and Measures for Key State-monitored Enterprise Supervisory Monitoring and Information Disclosure (Trial); Measures on Environmental Management and the Registration of Hazardous Chemicals (Trial); Measures on Open Environmental Information (Trial).

Clean Product Audit Information (4 pts): This category rates the disclosure of the mandated cleaner production audit enterprise list, as well as the status of whether enterprises have released their key pollutant emissions. If the enterprises failed to disclose this data, PITI rates whether or not the EPB has released the key pollution emission data for enterprises.

The 4 pts are divided into 4 parts: systematicness (1 pts), timeliness (1 pts), completeness (1 pts), and user-friendliness (1 pts). The level of systematicness is mainly determined by how many clean product audit firms are published compared to the total number of clean product firms and whether the firms or local EPBs publish the emission information of the firms (e.g., 1 pts if more than half of the firms are published and more than 2/3 firms released their emission information; 0.8 pts if less than 1/2 firms are published and more than 2/3 firms released their emission information; etc.). The level of timeliness is according to the time difference between the creation and publication of the relevant document or report (e.g., 1 pts if the document is published within one month after the document is created; 0.8 pts if the time difference is longer than one month and within three months;). The completeness is a measure of how much critical information firms or local EPBs publish (e.g., +0.2 pts if there is a firm list; +0.2 if there is information about the use of toxic and hazardous chemicals; etc.). The level of user-friendliness is determined by whether the information on the clean product audit is provided in a format that is convenient for the public (e.g., +0.2 pts if there is a website search engine; +0.6 pts if there is a column or page specifically for this information).

Principal Laws or Regulations: Provisional Measures for Clean Production Audit; Notification Concerning the Reinforcement of Pollution Source Environmental Supervisory Information Disclosure.

Environmental Impact Assessment Information.

Environmental Impact Assessment Information (15 pts): This category rates the disclosure of the full text of EIA reports, as well as the level of effort made by the EPBs to gather public opinions and notify interested parties of their rights to administrative reconsideration and administrative litigation through media channels, community assemblies, public hearings, or other methods, which should be undertaken before there is an acceptance or rejection of any construction project's EIA.

The 15 pts are divided into 4 parts: systematicness (5 pts), timeliness (4 pts), completeness (3 pts), and user-friendliness (3 pts). The level of systematicness is measured by the ratio of the number of local EIA projects whose EIA report are published (b) to the total number of project that require an EIA (a) (e.g., 5 pts if b/a is 1; 4 pts if b/a is larger or equal to 4/5 and smaller than 1; etc.). The level of timeliness is determined by whether the local EPBs widely notified the public about pending EIAs through a variety of media at the beginning of the EIA process and during the time when public opinions are solicited (e.g., +0.8 pts if there is a wide notice; +0.8 if there is a EIA public hearings; etc.). The level of completeness depends on how much critical information is available (e.g., +0.6 pts if there is a mention of the involved community; +0.6 pts if there are specific regulations or laws that the firm violated and specific emission standards; +1.6 pts if there are monitoring conclusion regarding compliance with EIAs). The level of user-friendliness is determined by whether the information on EIAs is provided in a format that is convenient for the public (e.g., +0.6 pts if there is a column or page specifically for the relevant information; +0.6 pts if there is an announcement of significant potential impacts through social media; etc.).

Principal Laws or Regulations: Notification to Issue the Construction Projects' Environmental Impact Assessment Government Information Disclosure Guidelines (Trial); Measures on Open Environmental Information (Trial); Provisional Measures for Public Participation throughout the Environmental Impact Assessment Process for Construction Projects.

Note on year-to-year changes. In some years, IPE changes the weighting of the PITI index to match the evolving legal status of transparency rules for municipal governments. In our case, the baseline 2015 index (rating 2014 performance) and the post-treatment 2016/17 index (rating 2015/16 performance) are slightly different. In particular, the disclosure of "Non-state-controlled Key Pollution Sources" is a new item in the second-year evaluation, with a weight of 6 pts. Accordingly, the weight of "Key Enterprises Data" decreased from 16pts to 12pts, and the weight of "Clean Product Audit Information" decreased from 4pts to 2pts.

Non-state-controlled Key Pollution Sources (6 pts): Disclosure of a list of non-state-controlled key pollution sources required by the newly revised Environmental Protection Law issued in January 2015. Disclosure of monitoring data of non-state-controlled air pollution enterprises.

Principal Laws or Regulations: Law of the People's Republic of China on the Prevention and Control of Atmospheric Pollution; Measures for the Disclosure of Environmental Information by Enterprises and Public Institutions; Measures on Self-Monitoring and Information Disclosure of Key State Monitored Enterprises (Trial), and Measures for Key State-monitored Enterprise Supervisory Monitoring and Information Disclosure (Trial); Environmental Protection Law of the People's Republic of China.

We produced both the baseline 2015 and post-treatment 2016/17 index scores for each of the post-treatment year reported in this paper. We use the consistent baseline 2015 index scoring for all analyses. We released the post-treatment 2016/17 index score in partnership with IPE for each of the post-treatment years.

PITI Scoring Process. We collected primary data from the websites of municipal Environmental Protection Bureaus. After data collection, six trained evaluators blinded to the treatment assignment of each city conducted assessments of transparency for each municipality in accordance with IPE's guidelines for assigning the PITI score. It takes approximately one person-week to collect and process data into a city PITI score. Every evaluator was asked to make a detailed record during the evaluation process, including data sources, scoring reasons, and decisions on all sub-scores. We applied a cross-check procedure within our research team and IPE further validated the rating in pre- and post-treatment waves of data collection.

To evaluate responsiveness to public information requests, we made a public request without revealing the reason for the request and evaluated the responsiveness of the EPB to that request on the basis of how systematic it was, timeliness, completeness, and friendliness. In the first-year evaluation, we requested all written decisions of administrative penalties in the third quarter of 2014 and a list of environmental impact assessment reports in the third quarter of 2014. In the second-year evaluation, we requested the number of written decisions on administrative penalties in the whole year of 2015 and a list of environmental impact assessments that involved public hearings. In the third-year evaluation, we requested the number of environmental impact assessments, a list of environmental impact assessments that involved public hearings, and the number of written decisions on administrative penalties in the whole year of 2016. Every year, we used the application form provided by IPE.

D. Dissemination of PITI Treatment

Online Publication. Every year in September to November, IPE published their report through two main new media platforms, WeChat and Weibo, which are the two most influential social platforms in China. The scores of the treated cities from our research team were included in the appendix of the IPE primary report in 2015, 2016, and 2017. Following this, we published the 2014, 2015, and 2016 PITI scores of the 25 treated cities through WeChat and Weibo in September 2015, November 2016, and November 2017 respectively. A number of other organizations forwarded our PITI report once it was released in these online platforms.

Publication by IPE. IPE mentioned our evaluation several times in their primary report and they sent their report to local Environmental Protection Bureaus after the online publications. In addition, IPE also published our PITI report on both Wechat and Weibo.

Communication with EPBs. After online publication, we sent our specific PITI report as well as IPE's primary report to the Environmental Protection Bureaus in the 25 treated municipalities to inform them of their PITI scores directly. We called them a week later to ensure that they had received the reports.

E. Main Results in Tabular Format and Additional Results

Table S3. Effects of Transparency Improvement on City-level Violations

	(1)<br>log(Number of<br>Firm-Day Violations)	(2)<br>Whether any<br>Firms Violate
$Treat \times Post$	-.469	-.14*
	(.416)	(.079)
Control Mean	16.4	.508
Control SD	48.8	.5
Month FE	Yes	Yes
City FE	Yes	Yes
Observations	3600	3600
R2	.465	.372

NOTES: This table shows the regression results from estimating Equation (1). Standard errors clustered at the city level are in parentheses. * $p < 0.1$, ** $p < 0.05$, *** $p < 0.01$

Table S4. Effects of Transparency Improvement on City-level Violations by Outlet

	(1) log(Number of Outlet-Day Violations)	(2) Whether any Outlets Violate
$Treat \times Post$	-.501	-.135*
	(.453)	(.0792)
Control Mean	20.1	.484
Control SD	59.6	.5
Month FE	Yes	Yes
City FE	Yes	Yes
Observations	3600	3600
R2	.471	.375

NOTES: This table shows the regression results from estimating Equation (1) using outlet-level data. Standard errors clustered at the city level are in parentheses. * $p < 0.1$, ** $p < 0.05$, *** $p < 0.01$

After our experimental intervention, several local NGOs began to score and publish PITI for cities in their province. The majority of the cities involved in our experiment did not receive additional intervention, and, as of 2019, only six cities in Hubei Province (three in the treatment group and three in the control group) have been assessed by a local NGO. For the analysis in the main text, we always use the assigned treatment as the independent variable in the analysis, for an intent-to-treat analysis. For robustness checks, we exclude the data of these six cities after 2019 as well as include them but assign them to treatment group after 2019 as robustness checks.

Table S5. Effects of Transparency Improvement on City-level Violations using Alternative Samples

	(1)<br>log(Number of<br>Firm-Day Violations)	(2)<br>Whether any<br>Violating Firms
Panel A. As treated		
$Treat \times Post$	-.813***	-.239***
	(.302)	(.0827)
Control Mean	17	.514
Control SD	49.7	.5
Month FE	Yes	Yes
City FE	Yes	Yes
Observations	3,600	3,600
R2	.474	.379
Panel B. Dropping later treated		
$Treat \times Post$	-.588	-.168**
	(.411)	(.0773)
Control Mean	17	.514
Control SD	49.7	.5
Month FE	Yes	Yes
City FE	Yes	Yes
Observations	3,456	3,456
R2	.496	.393

NOTES: This table shows the regression results from estimating Equation (1). Panel A reports the results by regarding samples from Hubei Province collected after 2019 as treatment group. Panel B presents the results after removing samples from Hubei Province after 2019. We use city-month level data. Standard errors clustered at the city level are in parentheses. * $p < 0.1$, ** $p < 0.05$, *** $p < 0.01$

Table S6. Effects of Transparency Improvement on Air Quality

	City level			Station level		
	(1a)	(2a)	(3a)	(1b)	(2b)	(3b)
	PM2.5	PM10	AQI	PM2.5	PM10	AQI
$Treat \times Post$	-3.7*	-6.14*	-4.77*	-3.71*	-5.1	-4.56*
	(2)	(3.51)	(2.38)	(2.04)	(3.43)	(2.43)
Control Mean	38.4	67.8	62.9	39.5	70.8	65
Control SD	20.3	32.5	25.6	21	33.7	26.2
Month FE	Yes	Yes	Yes	Yes	Yes	Yes
City FE	Yes	Yes	Yes			
Station FE				Yes	Yes	Yes
Observations	3600	3600	3600	13010	13009	13010
R2	.736	.769	.765	.723	.756	.753

NOTES: This table shows the regression results from estimating Equation (1). In columns (a), we use city-month level data. In columns (b), we use station-month level data. Standard errors clustered at the city level are in parentheses. * $p < 0.1$, ** $p < 0.05$, *** $p < 0.01$.

Table S7. Effects of Transparency Improvement on Air Quality using Alternative Samples

	City level			Station level		
	(1a)	(2a)	(3a)	(1b)	(2b)	(3b)
	PM2.5	PM10	AQI	PM2.5	PM10	AQI
Panel A. As treated						
$Treat \times Post$	-3.81**	-7.09***	-4.92***	-3.79**	-5.85**	-4.59**
	(1.55)	(2.3)	(1.67)	(1.58)	(2.36)	(1.74)
Control Mean	38.2	67.8	62.8	39.3	70.9	64.9
Control SD	20.2	32.8	25.6	20.9	34	26.2
Month FE	Yes	Yes	Yes	Yes	Yes	Yes
City FE	Yes	Yes	Yes			
Station FE				Yes	Yes	Yes
Observations	3,600	3,600	3,600	13,010	13,009	13,010
R2	.736	.769	.765	.723	.757	.753
Panel B. Dropping later treated						
$Treat \times Post$	-3.72*	-6.41*	-4.85**	-3.74*	-5.26	-4.61*
	(1.94)	(3.42)	(2.32)	(1.97)	(3.36)	(2.36)
Control Mean	38.2	67.8	62.8	39.3	70.9	64.9
Control SD	20.2	32.8	25.6	20.9	34	26.2
Month FE	Yes	Yes	Yes	Yes	Yes	Yes
City FE	Yes	Yes	Yes			
Station FE				Yes	Yes	Yes
Observations	3,456	3,456	3,456	12,483	12,483	12,483
R2	.738	.77	.768	.725	.758	.757

NOTES: This table shows the regression results from estimating Equation (1).Panel A reports the results by regarding samples from Hubei Province collected after 2019 as treatment group. Panel B presents the results after removing samples from Hubei Province after 2019. In Column (a), we use city-month level data. In Column (b), we use station-month level data. Standard errors clustered at the city level are in parentheses. * $p < 0.1$, ** $p < 0.05$, *** $p < 0.01$.

Table S8. Effects of Transparency Improvement on Firm-level Violations

	(1) log (Number of Violations) Top 10%	(2) log (Number of Violations) Top 25%	(3) log (Number of Violations) Mean
$High \times Treat \times Post$	-.826***	-.396*	-.335*
	(.28)	(.207)	(.197)
$Treat \times Post$	.163	.012	-.0693
	(.221)	(.106)	(.078)
$High \times Post$	-1.71***	-1.95***	-1.92***
	(.225)	(.115)	(.0973)
Month FE	Yes	Yes	Yes
Firm FE	Yes	Yes	Yes
Observations	21,991	21,991	21,991
R2	.395	.452	.459

NOTES: This table shows the regression results using firm-month level data. High violation groups are identified as the top 10%, top 25%, and above mean of benchmark violating firms in columns (1), (2), and (3). Standard errors clustered at the city level are in parentheses. * $p < 0.1$, ** $p < 0.05$, *** $p < 0.01$

Table S9. Effects of Transparency Improvement on Firm-level Violations (Binary)

	(1) Whether Violate Top 10%	(2) Whether Violate Top 25%	(3) Whether Violate Mean
$High \times Treat \times Post$	-.277**	-.146*	-.126*
	(.109)	(.074)	(.0653)
$Treat \times Post$	.0675	.0344	.013
	(.0879)	(.0629)	(.0554)
$High \times Post$	-.344***	-.481***	-.494***
	(.0861)	(.0484)	(.0374)
Month FE	Yes	Yes	Yes
Firm FE	Yes	Yes	Yes
Observations	21,991	21,991	21,991
R2	.263	.286	.291

NOTES: This table shows the regression results using firm-month level data. High violation groups are identified as the top 10%, top 25%, and above mean of benchmark violating firms in columns (1), (2), and (3). Standard errors clustered at the city level are in parentheses. * $p < 0.1$, ** $p < 0.05$, *** $p < 0.01$

Table S10. Effects of Transparency Improvement on Firm-level Violations using Alternative Samples

	(1) log (Number of Violations) Top 10%	(2) log (Number of Violations) Top 25%	(3) log (Number of Violations) Mean
Panel A. As treated			
$High \times Treat \times Post$	-.943***	-.471**	-.404**
	(.256)	(.211)	(.18)
$Treat \times Post$	.135	.0303	-.000972
	(.152)	(.0765)	(.0659)
$High \times Post$	-1.63***	-1.91***	-1.87***
	(.209)	(.118)	(.102)
Month FE	Yes	Yes	Yes
Firm FE	Yes	Yes	Yes
Observations	21,991	21,991	21,991
R2	.397	.453	.459
Panel B. Dropping later treated			
$High \times Treat \times Post$	-.857***	-.425**	-.357*
	(.287)	(.204)	(.195)
$Treat \times Post$	.15	.00198	-.0785
	(.221)	(.106)	(.0801)
$High \times Post$	-1.66***	-1.93***	-1.9***
	(.232)	(.115)	(.0991)
Month FE	Yes	Yes	Yes
Firm FE	Yes	Yes	Yes
Observations 21,061	21,061	21,061	
R2	.407	.466	.472

NOTES: This table shows the regression results using firm-month level data. Panel A reports the results by regarding samples from Hubei Province collected after 2019 as treatment group. Panel B presents the results after removing samples from Hubei Province after 2019. High violation groups are identified as the top 10%, top 25%, and above mean of benchmark violating firms in columns (1), (2), and (3). Standard errors clustered at the city level are in parentheses. * $p < 0.1$, ** $p < 0.05$, *** $p < 0.01$

Table S11. Effects of Transparency Improvement on Firm-level Violations (Binary) using Alternative Samples

	(1) Whether Violate Top 10%	(2) Whether Violate Top 25%	(3) Whether Violate Mean
Panel A. As treated			
$High \times Treat \times Post$	-.325***	-.19***	-.172***
	(.091)	(.0644)	(.0526)
$Treat \times Post$	.0574	.0389	.0349
	(.0616)	(.0435)	(.0394)
$High \times Post$	-.312***	-.46***	-.47***
	(.0802)	(.043)	(.0334)
Month FE	Yes	Yes	Yes
Firm FE	Yes	Yes	Yes
Observations	21,991	21,991	21,991
R2	.264	.287	.292
Panel B. Dropping later treated			
$High \times Treat \times Post$	-.289**	-.155**	-.134**
	(.111)	(.0734)	(.0649)
$Treat \times Post$	.0646	.0325	.0117
	(.0877)	(.0625)	(.0553)
$High \times Post$	-.326***	-.471***	-.484***
	(.0886)	(.047)	(.0363)
Month FE	Yes	Yes	Yes
Firm FE	Yes	Yes	Yes
Observations	21,061	21,061	21,061
R2	.271	.294	.3

NOTES: This table shows the regression results using firm-month level data. Panel A reports the results by regarding samples from Hubei Province collected after 2019 as treatment group. Panel B presents the results after removing samples from Hubei Province after 2019. High violation groups are identified as the top 10%, top 25%, and above mean of benchmark violating firms in columns (1), (2), and (3). Standard errors clustered at the city level are in parentheses. * $p < 0.1$, ** $p < 0.05$, *** $p < 0.01$

Table S12. Average Treatment Effect on Environmental Inspections

	(1) log(Number of Inspections) Include 0 Values	(2) log(Number of Inspections) Exclude 0 Values
Treat	.643**	.599**
	(.288)	(.242)
Control Mean	117	151
Control SD	245	269
Month FE	Yes	Yes
Block FE	Yes	Yes
Observations	2,400	1,911
R2	.466	.504

NOTES: This table shows the regression results using city-level data from 2017 to 2020. Column (1) includes 0 values of number of inspections. Column (2) excludes 0 values of number of inspections. Standard errors clustered at the city level are in parentheses. * $p < 0.1$, ** $p < 0.05$, *** $p < 0.01$

To address the possibility that the reductions to ambient air pollution are a result of data manipulation, we assess the robustness of those results to measuring air pollution using Aerosol Optical Thickness, which characterizes how light is absorbed and scattered in the atmosphere. The PM10/PM2.5 data we used is sourced from the ChinaHighAirPollutants (CHAP) dataset, which is available through National Earth System Science Data Center (https://nnu.geodata.cn/featured_data.html). This dataset is derived from MODIS satellite data, with a spatial resolution of 10 km, which was then resampled to 1 km using bilinear interpolation for higher spatial accuracy (2, 3). Based on this data, we calculated the monthly average PM10/PM2.5 values for each city by averaging all 1 km grid data within the city boundaries over the course of a month.

The authors of this dataset did not explicitly isolate or remove dust contributions in the estimation of PM concentrations. While natural sources like dust can contribute to aerosol optical thickness levels, the key concern in our study is the dynamic change in pollution levels within different cities, using a difference-in-differences model with city fixed effects. This approach accounts for the influence of any persistent city-specific characteristics, such as persistent natural dust sources. Additionally, since our estimates are based on comparing differences in dynamic changes across experimental groups, any influence of dust should be balanced in expectation. These results are also secondary and consistent with data from on-ground ambient air pollution monitors and in-stack industrial facility monitors. The latter cannot be affected by dust. As displayed in Table S13, the results are consistent using this alternative measure.

Table S13. Average Treatment Effect on Air Quality using Satellite Data

	(1)<br>PM2.5	(2)<br>PM10
$Treat \times Post$	-3.63**	-4.39*
	(1.48)	(2.5)
Control Mean	37.5	65.8
Control SD	17.6	28.9
Month FE	Yes	Yes
City FE	Yes	Yes
Observations	4,200	4,200
R2	.813	.847

NOTES: This table shows the regression results from estimating Equation (1) using data from 2014 to 2020. Standard errors clustered at the city level are in parentheses. * $p < 0.1$, ** $p < 0.05$, *** $p < 0.01$.

Table S14. Average Treatment Effect on Firm-level Abnormal Emissions Data

	(1)<br>log(Abnormal Days where<br>Operated less than20 Hours in a Day)	(2)<br>log(Abnormal Days with<br>Abnormally Low Recorded Concentration)
$High \times Treat \times Post$	.0474	.119
	(.0694)	(.0767)
$Treat \times Post$	.03	-.0342
	(.0493)	(.0423)
$High \times Post$	.0638	-.0795
	(.0574)	(.0645)
Month FE	Yes	Yes
Firm FE	Yes	Yes
Observations	21,991	21,991
R2	.143	.145

NOTES: This table shows the regression results using firm-month level data. Abnormal Hour is a dummy variable that equals 1 if the firm's hourly records are fewer than 20 on that day, and zero otherwise. Abnormal Concentration is a dummy variable that equals 1 if the firm's daily average emission concentration is smaller than 1/10 annual average level, and zero otherwise. Standard errors clustered at the city level are in parentheses. * $p < 0.1$, ** $p < 0.05$, *** $p < 0.01$

Table S15. Balance Test on City Characteristics

Variable	Treatment Mean (SD)	Control Mean (SD)	Difference (p-value)
GDP per capita	45461.52	41380.60	4080.92
	(15957.76)	(18656.04)	(0.41)
Population	379.37	349.59	29.784
	(185.53)	(187.58)	(0.58)
Secondary industry in GDP	51.04	48.18	2.856
	(9.01)	(10.12)	(0.30)
Fiscal expenditure	23360.04	21495.73	1864.3128
	(8461.12)	(8497.60)	(0.44)
Fiscal revenue	8530.72	6067.24	2463.48
	(7741.27)	(4878.31)	(0.18)

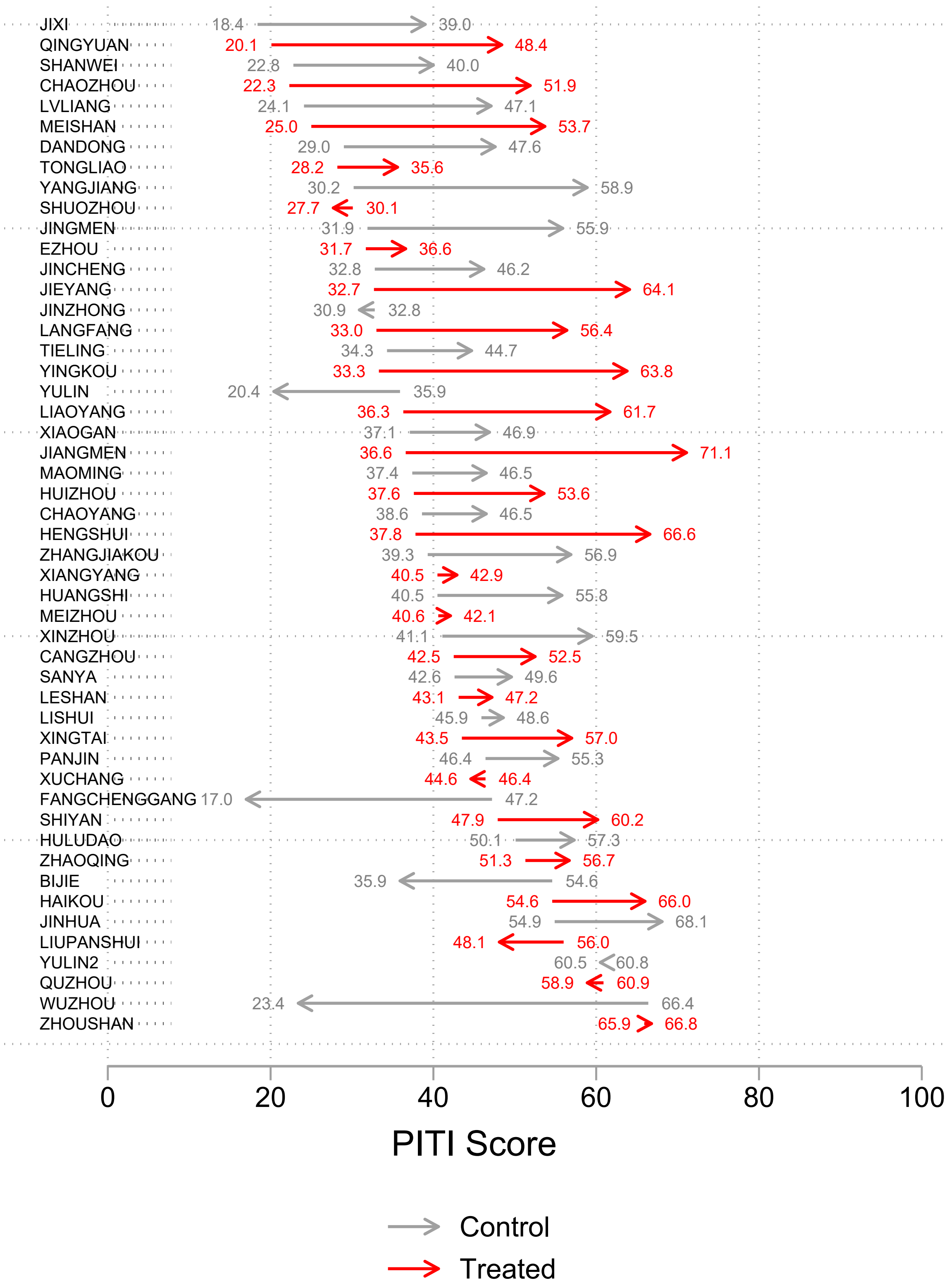

Fig. S1. PITI Score Changes of the 50 Cities from 2014 to 2016

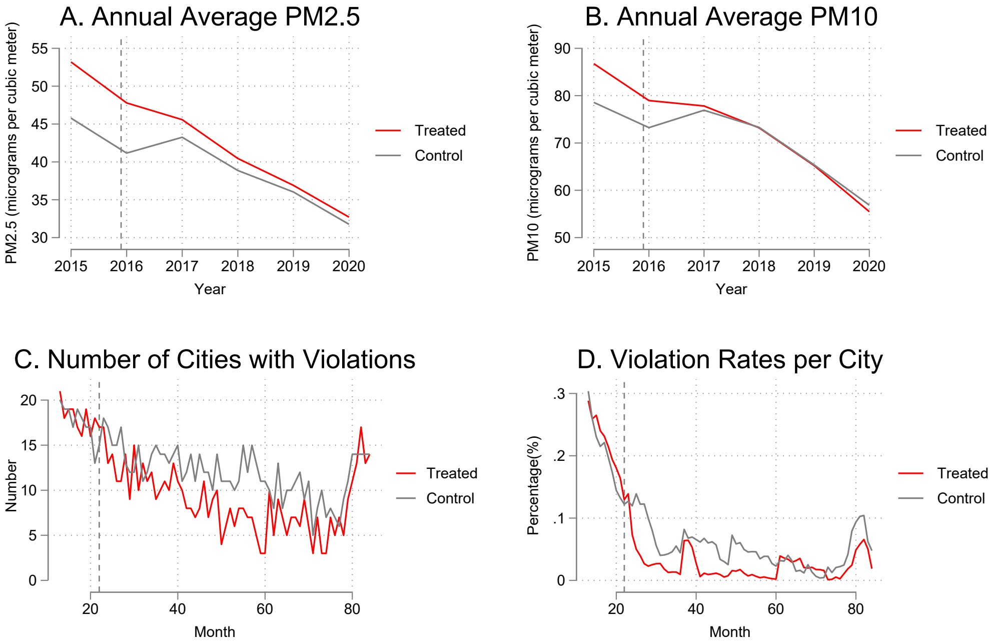

Fig. S2. Summary of Air Quality Changes and Violation Changes

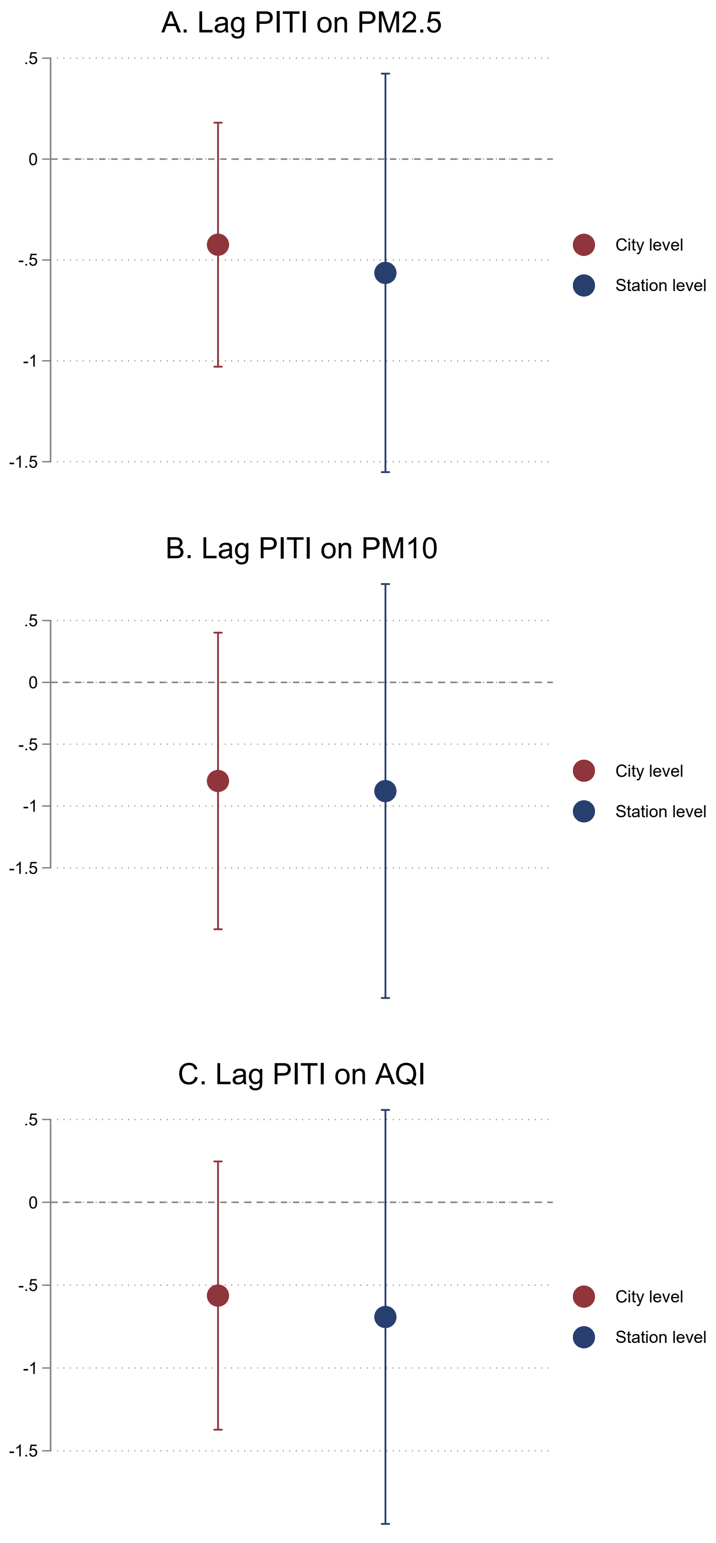

Fig. S3. Effects of PITI on Air Quality *Notes*: This figure reports the IV results of transparency on air quality from 2015 to 2017, using the treatment as the instrumental variable. Notably, PITI only contains annual values, thus we used imputation method to calculate monthly values. Because the PITI data only covers three years, we are unable to observe longer-term effects. This limitation is particularly relevant since our baseline regression indicates that the impact of increased transparency on environmental governance takes time to appear. In all regressions we use monthly-city data, control for month fixed effects and city/station fixed effects, and cluster standard errors at the city level.

We focus on the indicator of whether firms exit from the database. We use two non-parametric estimation methods (Kaplan-Meier survival estimate and Nelson-Aalen cumulative hazard estimate) to analyze whether the intervention of information disclosure leads to more firm exits. The Kaplan-Meier survival curve offers a step function, providing a survival probability at each time point. In contrast, the Nelson-Aalen cumulative hazard estimate focuses on the cumulative risk of exiting, summing up the hazard rates at each time point, displaying the cumulative exit risk up to each time point.

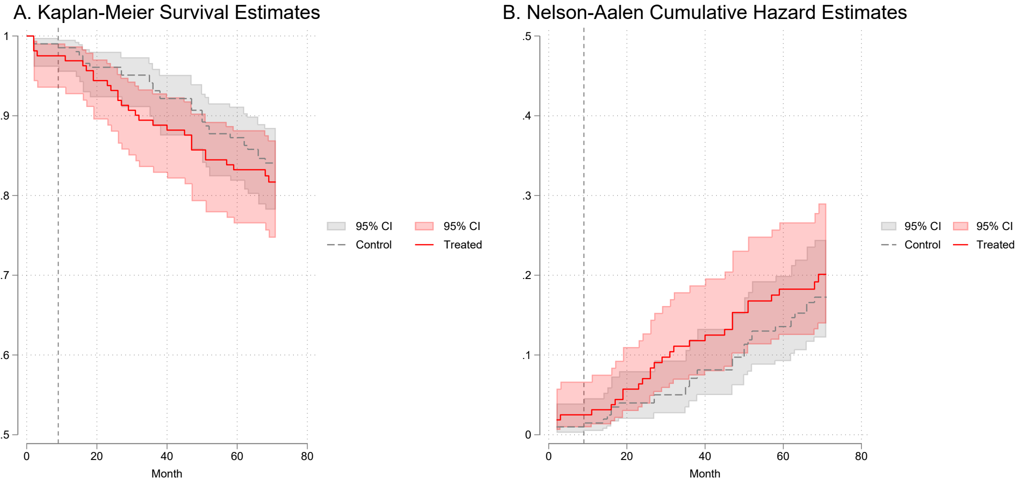

Fig. S4. Effects of the Transparency Intervention on Firm Exit *Notes*: This figure reports the survival analysis and exit hazard of high polluting enterprises in the treatment group and the control group.

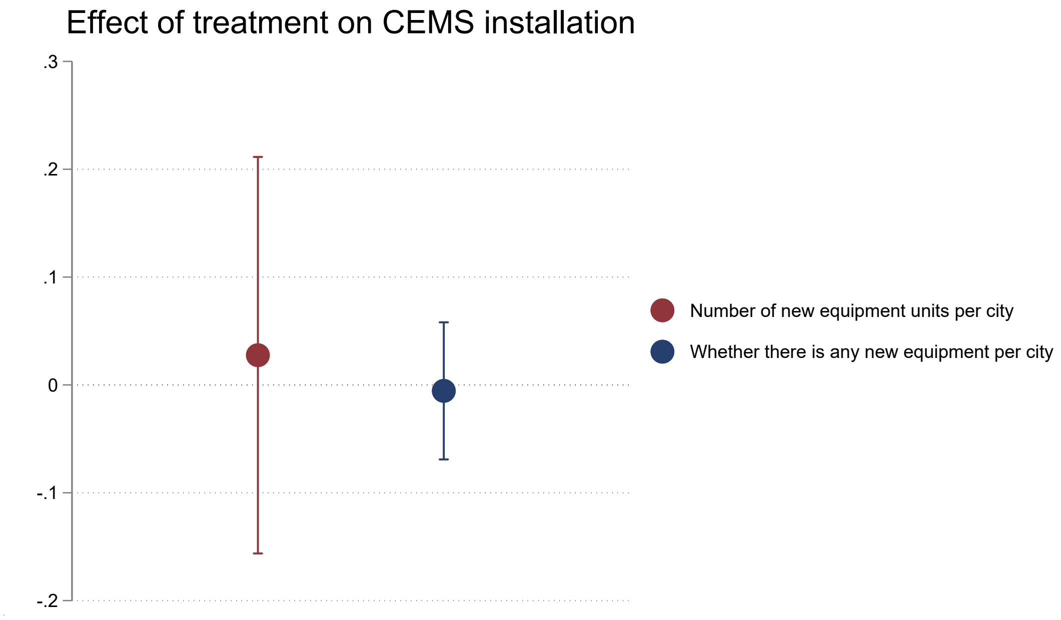

Fig. S5. Effects of the Transparency Intervention on Installation of CEMS Equipment in Existing Firms *Notes*: This figure reports the impact of being rated by the PITI program on number of new installation of CEMS equipment in existing firms and whether any new installation of CEMS equipment in existing firms from 2015 to 2020. The figure presents coefficients and 90% confidence intervals on Treat*Post from estimating Equation (1). We use monthly-city data, control for month fixed effects and city fixed effects, and cluster standard errors at the city level in all specifications.

272 **F. Sample Characteristics**

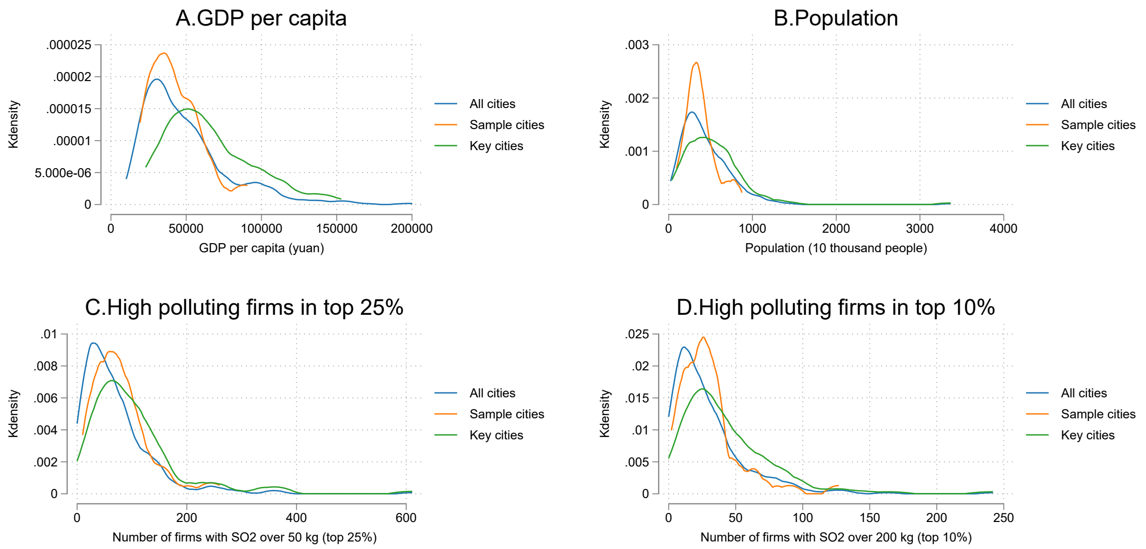

Fig. S6. Characteristics of Our Sample Cities and All Cities *Notes*: This figure reports the distribution of GDP per capita and populations of our sample cities, all cities, and key cities in 2014.

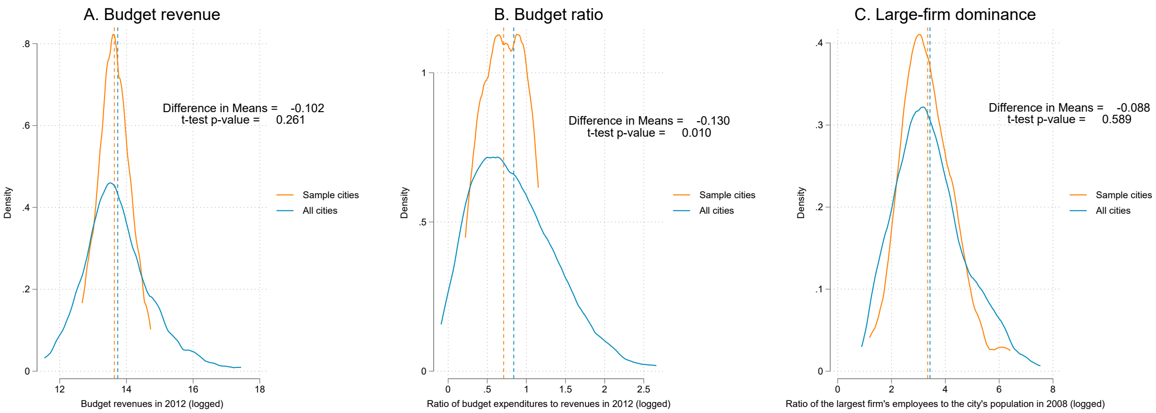

Fig. S7. Characteristics of our sample cities and all cities on criteria used to select the sample *Notes*: This figure reports the distribution of budget revenue, dependence on central transfers, and large firm dominance in sample cities and all cities.

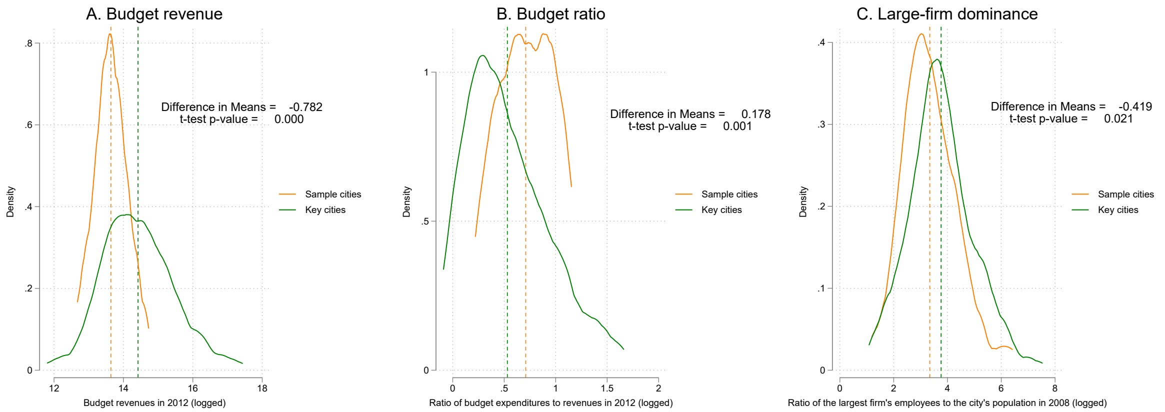

Fig. S8. Characteristics of our sample cities and key cities on criteria used to select the sample *Notes*: This figure reports the distribution of budget revenue, dependence on central transfers, and large firm dominance in sample cities and key cities for pollution control, which were previously rated using PITI by IPE.

G. Potential Confounding Policies

One concern is that the environmental policies implemented after the treatment could have differed in intensity between the treatment and control groups, potentially confounding our results. To address this concern, we conducted a balance test on the two most important environmental policies in China from 2015 to 2020 for both the treatment and control groups.

The "Three-Year Action Plan to Win the Blue Sky Defense War" (2018) is an ambitious environmental policy introduced by China to significantly improve air quality nationwide. This plan sets specific targets and measures to combat air pollution from 2018 to 2020, focusing on three key regions crucial for national air quality improvement: Beijing-Tianjin-Hebei and Surrounding Areas: Includes the cities of Beijing, Tianjin, and surrounding cities in Hebei Province; Yangtze River Delta Region: Encompasses Shanghai, Jiangsu, Zhejiang, and Anhui provinces; Fen-Wei Plain: Includes cities in Shanxi, Shaanxi, and Henan provinces. In our sample, there are 8 cities in the treatment group and 8 cities in the control group that belong to these three key regions, making the distribution essentially balanced.

The Central Environmental Protection Inspectorate is another key initiative by the Chinese government to strengthen environmental governance and ensure compliance with environmental laws and regulations. Launched in 2016, this initiative involves periodic inspections of provinces, autonomous regions, and municipalities to assess their adherence to environmental policies and standards. We conducted a balance test to examine the distribution of treatment and control groups across different inspection batches. The results show no significant difference between the groups, with a Chi-square value (Chi2) of 6.746 and a p-value of 0.149, indicating that there is no evidence of a distribution inconsistent with balanced groups.

Table S16. Distribution of cities across inspection batches

Batch	Treated: 25 cities	Control: 25 cities
Jan-16	4	1
Jul-16	2	4
Nov-16	10	7
Apr-17	4	10
Aug-17	5	3

H. Health Effects Estimation

Our estimates suggest that the transparency treatment reduced PM2.5 by 3.7 micrograms per cubic meter in the treated cities, a 9.6% drop from baseline levels. We conducted a back-of-the-envelope calculation of the resulting health benefits. Current research shows that a 10 microgram decrease in PM2.5 reduces daily all-cause mortality by 0.68% (95% CI, 0.59–0.77), cardiovascular mortality by 0.55% (95% CI, 0.45–0.66), and respiratory mortality by 0.74% (95% CI, 0.53–0.95) (4). This implies that each 1 microgram reduction in PM2.5 would lower these rates by 0.068%, 0.055%, and 0.074%, respectively. Therefore, the 3.7 microgram reduction in PM2.5 from the transparency treatment is estimated to decrease all-cause mortality by 0.25% (95% CI, 0.22–0.28), cardiovascular mortality by 0.2% (95% CI, 0.17–0.24), and respiratory mortality by 0.27% (95% CI, 0.2–0.35). With an initial all-cause mortality of 803,110 annually in the 25 treated cities, this reduction translates to 2,008 fewer deaths per year. Extrapolating this effect nationwide, where annual all-cause mortality was 9.74 million, similar reductions in PM2.5 could prevent 24,350 deaths across China each year.

References

1. SE Anderson, MT Buntaine, M Liu, B Zhang, Non-governmental monitoring of local governments increases compliance with central mandates: a national-scale field experiment in china. *Am. J. Polit. Sci.* **63**, 626–643 (2019).
2. J Wei, et al., Improved 1 km resolution pm2.5 estimates across china using enhanced space–time extremely randomized trees. *Atmospheric Chem. Phys.* **20**, 3273–3289 (2020).
3. J Wei, et al., Reconstructing 1-km-resolution high-quality pm2. 5 data records from 2000 to 2018 in china: spatiotemporal variations and policy implications. *Remote. Sens. Environ.* **252**, 112136 (2021).
4. C Liu, et al., Ambient particulate air pollution and daily mortality in 652 cities. *New Engl. J. Medicine* **381**, 705–715 (2019).
